# Glucocorticoids induce a phagocytic C1Q+ macrophage phenotype primed for IFNγ-dependent CXCL9 secretion

**DOI:** 10.1038/s41598-026-52733-y

**Published:** 2026-05-18

**Authors:** Alexandra S. Triebig, Tanja Maier, Paul Schwarzlmueller, Sena Atici, Martin E. Kirmaier, Vasileios Chortis, Kleiton S. Borges, David T. Breault, Claudio Ribeiro, Adrian Gottschlich, Laura-Sophie Landwehr, Nicole Bechmann, Mirko Peitzsch, Nagarajan Paramasivam, Sebastian Theurich, Stefan Fröhling, Hanno Glimm, Daniel Hübschmann, Martin Fassnacht, Martin Reincke, Jose Pedro Friedmann-Angeli, Isabel Weigand, Matthias Kroiss

**Affiliations:** 1 Department of Medicine IV, LMU University Hospital, LMU Munich, Ziemssenstr. 5, Munich, 80336 Germany; 2 Rudolf Virchow Center for Experimental Biomedicine, University of Würzburg, Würzburg, Germany; 3 Department of Medicine III, LMU University Hospital, LMU Munich, Munich, Germany; 4 Cancer and Immunometabolism Research Group, Gene Center, LMU Munich, Munich, Germany; 5 Division of Endocrinology, Boston Children’s Hospital, Boston, MA 02115 USA; 6 Department of Pediatrics, Harvard Medical School, Boston, MA 02115 USA; 7 Division of Clinical Pharmacology, LMU University Hospital, LMU Munich, Munich, Germany; 8 Bavarian Cancer Research Center (BZKF), Munich/Würzburg, Germany; 9 German Cancer Consortium (DKTK), a partnership between LMU Hospital and DKTK Heidelberg, Munich, Germany; 10 Department of Internal Medicine I, Division of Endocrinology and Diabetes, University Hospital, University of Würzburg, Würzburg, Germany; 11 Institute of Clinical Chemistry and Laboratory Medicine, Medical Faculty Carl Gustav Carus, University Hospital Carl Gustav Carus, TUD Dresden, Dresden, Germany; 12 Computational Oncology Group, Molecular Precision Oncology Program, National Center for Tumor Diseases (NCT), NCT Heidelberg, a partnership between the German Cancer Research Center (DKFZ) and Heidelberg University Hospital, Heidelberg, Germany; 13 Division of Translational Medical Oncology, German Cancer Research Center (DKFZ), Heidelberg, Germany; 14 National Center for Tumor Diseases (NCT), NCT Heidelberg, a partnership between DKFZ and Heidelberg University Hospital, Heidelberg, Germany; 15 German Cancer Consortium (DKTK), Core Center Heidelberg, Heidelberg, Germany; 16 Division of Translational Precision Medicine, Institute of Human Genetics, Heidelberg University, Heidelberg, Germany; 17 Department for Translational Medical Oncology, , National Center for Tumor Diseases (NCT/UCC) Dresden, Faculty of Medicine and University Hospital Carl Gustav Carus, TUD Dresden University of Technology, Helmholtz-Zentrum Dresden-Rossendorf (HZDR), Dresden, Germany; 18 Translational Medical Oncology, Faculty of Medicine and University Hospital Carl Gustav Carus, TUD Dresden University of Technology, Dresden, Germany; 19 German Cancer Consortium (DKTK), partner site Dresden, Dresden, Germany; 20 Pattern Recognition and Digital Medicine Group, Heidelberg Institute for Stem Cell Technology and Experimental Medicine (HI-STEM GmbH), Heidelberg, Germany; 21 Innovation and Service Unit for Bioinformatics and Precision Medicine, DKFZ, Heidelberg, Germany; 22 Comprehensive Cancer Center Mainfranken, University of Würzburg, Germany, and National Center for Tumor Diseases (NCT) WERA, Würzburg, Germany

**Keywords:** Adrenal cancer, Immune checkpoint inhibition, Tumor microenvironment, Macrophage polarization, Immunotherapy biomarker, Cancer, Immunology, Oncology

## Abstract

Glucocorticoids are an emerging component of the tumor microenvironment and play a multifaceted role in modulating immune responses. Adrenocortical carcinoma (ACC) is a rare endocrine malignancy characterized by systemic cortisol excess in ~ 60% of cases. Responses to immune checkpoint inhibitors (ICI) in patients with ACC are variable, and currently, no reliable biomarkers exist to predict therapeutic outcomes. Here, *in vitro-*differentiated and glucocorticoid-polarized peripheral blood monocytes from healthy donors were characterized using NanoString nCounter gene expression analysis, western blotting, and ELISA. *In vivo*, ACC-bearing mice were treated with immune checkpoint inhibitors and mifepristone. In human ACC tumor samples, immunohistochemistry was employed to quantify intratumoral macrophages, while mass spectrometry was applied to measure steroid hormone concentrations. We report that ACC tumors are highly infiltrated by CD68+/CD163 + macrophages, independent of cortisol overproduction. *In vitro*, glucocorticoid exposure polarized macrophages towards a C1Q+ CD163 + subtype with enhanced phagocytic activity. Upon IFNγ stimulation, secretion of the T cell chemoattractant CXCL9 was higher in C1Q+ macrophages than in classical pro-inflammatory M1 macrophages. In ACC-bearing mice, treatment with immune checkpoint inhibitors and concurrent blockade of the glucocorticoid receptor with mifepristone reduced tumoral CXCL9 expression and CD4 + T cell infiltration compared to ICI monotherapy. In ACC patient samples, intratumoral macrophage marker expression positively correlated with markers indicative of T cell infiltration and improved patient survival. Moreover, the percentage of macrophages detected by immunohistochemistry positively correlated with response to immunotherapy. Collectively, this study identifies a glucocorticoid-induced C1Q+ CD163 + macrophage phenotype that may contribute to T cell recruitment and enhance the efficacy of immunotherapy in ACC.

**Supplementary Information:**

The online version contains supplementary material available at 10.1038/s41598-026-52733-y.

## Introduction

Glucocorticoids (GCs) are a class of steroid hormones predominantly synthesized and secreted by the *zona fasciculata* of the adrenal cortex in response to hypothalamic-pituitary-adrenal (HPA) axis activation. Within cancer tissues, GCs have emerged as an important component of the tumor microenvironment (TME), originating from three main sources: endogenous production^1,2^, recycling of inactive metabolites^3^, or therapeutic administration. In the context of cancer, GCs are believed to promote tumor cell proliferation, survival, and migration while concurrently suppressing antitumor immunity through functional modulation of immune cells^1,4,5^.

Adrenocortical carcinoma (ACC) is a rare, yet highly aggressive tumor of the adrenal cortex, with an estimated annual incidence ranging from approximately 0.5 to 2.0 cases per million individuals^6,7^. While clinically non-functional ACCs show no signs of systemic cortisol excess and are commonly diagnosed incidentally or due to mass effects, the majority of ACCs are hormonally active, highlighting ACC as a valuable model for studying intra-tumoral GC dynamics. ACC is routinely treated with mitotane or mitotane-EDP (EDP-M). However, in both cases, patient survival remains poor, with an objective response rate to EDP-M of 23.2%, and a median progression-free survival of 5.0 months^8^. Immune checkpoint inhibitors (ICIs), which have profoundly changed the management of many malignancies, have demonstrated limited efficacy in ACC. So far, a combined analysis of twenty studies with a total of 250 patients reported an objective response in only 14% of patients with a median overall survival of 13.9 months^9^. However, a clinically meaningful overall response rate of 52% and a median overall survival (OS) of almost 21 months in one recent study suggests that a subset of patients may benefit from ICI therapy^10^. Nonetheless, a biomarker of response to ICI therapy is still lacking.

Tumor-associated macrophages (TAMs) exhibit a remarkable plasticity and can assume a dual role in the progression of cancer. Pro-inflammatory ‘M1-like’ (classically activated) macrophages (MΦ) fulfill antitumoral functions, including cytotoxicity and immune stimulation, e.g., by the release of pro-inflammatory cytokines. Meanwhile, anti-inflammatory ‘M2-like’ (alternatively activated) MΦ often promote tumor growth, fostering a profoundly immune-suppressed TME that negatively impacts cancer outcome^11,12^ or the responsiveness to ICI therapy^13^. Even though this simplified classification proposed by Mills *et al.* broadly describes the inflammatory capacity of MΦ, this terminology is considered outdated as the current understanding suggests a spectrum of diverse MΦ phenotypes, each with a unique contribution to inflammation^14,15^.

Therapeutic modulation of MΦ differentiation, such as inhibition of colony-stimulating factor 1 (CSF-1) or its receptor (CSF1R), has emerged as a promising approach to increase the efficacy of ICI in unresponsive or refractory tumors. However, clinical trials investigating CSF1R blockade, either as monotherapy or in combination with ICIs, have largely failed to produce significant clinical responses^16^. In ACC, therapeutic approaches targeting MΦ remain completely unexplored, primarily due to the limited understanding of MΦ infiltration and function in this rare endocrine malignancy. Current *in vitro* studies propose the activation of the glucocorticoid receptor (GR) and reprogramming of mitochondrial metabolism by GCs to profoundly suppress pro-inflammatory (M1) MΦ polarization and promote anti-inflammatory (M2) polarization^17,18^. The impact of tumor-derived GCs on the function of TAMs in cancer remains largely unknown.

ACC may serve as a useful model to study the role of GC activity in cancer and gain insights into the relevance of endogenously produced GCs across several malignancies. Here, we set out to identify the contribution of tumoral GCs to MΦ polarization in cancer and describe whether therapeutic targeting of the underlying mechanisms could be feasible.

## Materials and methods

### Tissue samples

Tissue samples from patients with ACC were collected as part of the European Network for the Study of Adrenal Tumors (ENSAT) registry study or the MASTER (Molecularly Aided Stratification for Tumor Eradication Research) program. The ENSAT study has been approved by the ethics committees of the Ludwig-Maximilians-University, Munich, Germany (RRID: SCR_011358) (approval number: 379/10) and the Julius-Maximilians-University Würzburg (approval number: 88/11). The MASTER program (ClinicalTrials.gov: NCT05852522), a prospective observational study conducted by the German Cancer Research Center (DKFZ), the National Center for Tumor Diseases (NCT), and the German Cancer Consortium (DKTK), leverages whole-genome/exome sequencing (WGS/WES), RNA sequencing (RNA-seq), DNA methylation profiling, proteomics, and phosphoproteomics to guide treatment in young adults with advanced malignancies and patients with incurable rare cancers^19^. This study was approved by the Ethics Committee of the Medical Faculty of Heidelberg University. All procedures involving human participants were conducted in accordance with the ethical principles outlined in the Declaration of Helsinki, and written informed consent was obtained from each patient prior to surgery.

### Immunohistochemistry

Immunohistochemistry (IHC) was conducted as described^20^. In short, formalin-fixed, paraffin-embedded (FFPE) tumor sections were deparaffinized in xylene and dehydrated with increasing dilutions of ethanol. Antigen retrieval was performed in 10 mM citric acid monohydrate buffer at pH 6.5. Endogenous peroxidase activity was blocked with methanol containing 3% H_2_O_2_ for 10 min at RT. Non-specific binding was blocked using 20% human AB serum for one hour at RT. Primary antibodies (suppl. Table 1) were applied overnight at 4 °C. Signals were developed using the HiDef Detection Polymer System Detection Amplifier (Medac, Germany) and HiDef Detection Polymer System HRP Polymer Detector (Medac, Germany). DAB (DAB Liquid Kit; Dako) was applied, and the sections were counterstained with hematoxylin. Stained slides were dehydrated in 100% EtOH and dried at 60 °C for one hour. Slides were scanned using the Microscopes International uScopeMXII-20 Digital Microscope (RRID: SCR_026399) with 20x magnification, and positive cell detection was performed using the software QuPath (RRID: SCR_018257).

### Human cell lines and drug treatment

NCI-H295R cells (RRID: CVCL 0458) were obtained from Cytion and cultured in DMEM/F12 supplemented with 1x Insulin-Transferrin-Selenium (Gibco) and 2.5% Nu-Serum (Corning). JIL-2266 cells were established and characterized in our laboratory and cultured as previously described^21^. All cell lines were cultured at 37 °C and 5% CO2. Cells were checked regularly for mycoplasma contamination using the Venor^®^ GeM Classic Mycoplasma Detection Kit (Minerva Biolabs). Ferroptosis was induced using RSL3 (Selleckchem). Inhibitors or antagonists (liproxstatin-1, metyrapone, or mifepristone (all Sigma)) were added one hour prior to cell death initiation, the start point of cell culture supernatant collection, or dexamethasone treatment, respectively. Dexamethasone (Jenapharm^®^) and hydrocortisone (Pfizer) were used as exogenous glucocorticoids. Cas9-expressing THP-1 (RRID: CVCL_0006) monocytes were obtained from Prof. Jörg Wischhusen, University Hospital Würzburg, and cultured in RPMI 10% FBS, 1% P/S, and 50 µM ß-mercaptoethanol. For differentiation, THP-1 cells were plated in 12-well plates and exposed to 10 ng/ml PMA for 48 h before polarizing factors were added for an additional 48 h. For *NR3C1* KO cells, THP-1 cells were first differentiated for two days using 10 ng/ml PMA, followed by detachment of cells using Accutase. Cells were counted, and 2.5*10^6 cells were transfected with 1.6 µg sgRNA (IDT) using the LONZA™ Nucleofector 2b Device (RRID: SCR_022262) (program V-001). After 5 h, the medium was changed to THP-1 medium containing 2.5 ng/ml PMA and 50 µM β-mercaptoethanol. 48 h after transfection, cytokines or CM were added for 48 h.

### Primary macrophage isolation and polarization

Human PBMCs from healthy blood donors were isolated by density gradient using Lymphoprep™ (StemCell) and transferred to 12-well plates at a density of 5*10^6 cells/ml in macrophage medium (RPMI-1640 (Gibco) supplemented with 10% FBS (Sigma), 1% penicillin-streptomycin (Sigma), and 1% L-glutamine (Gibco)). Monocytes attached to the culture vessel after 24 h were incubated in macrophage medium supplemented with either GM-CSF (Gibco; 100 ng/ml) or M-CSF (Gibco; 40 ng/ml) for ‘M1-like’ or ‘M2-like’ MΦ differentiation, respectively. On day 7, further polarizing factors IFNγ (Gibco; 10 ng/ml), IL-4 (Gibco; 20 ng/ml), LPS (Invitrogen; 100 ng/ml) or IL-10 (Gibco; 150 ng/ml) were added for a total of 6 days with medium change and PBS washing every other day. A schematic representation of the macrophage polarization protocol and respective phenotype characteristics are depicted in the supplementary Figure S8. For the generation of conditioned media (CM), ACC cell lines were cultured for 48 h at 3.5*10 × ^5 cells/well of a 6-well plate. Supernatant was collected, sterile-filtered, and mixed with macrophage medium (30% v/v). This medium was added to the monocytes from day 1 onwards. Medium was changed on day 6 and then every other day.

### Immunocytochemistry

PBMCs were isolated, and macrophages were differentiated and polarized as described above. For immunostaining, cells were fixed with 4% PFA for 10 min at 4 °C. Subsequently, cells were blocked with PBS/4% BSA for 30 min and incubated with the primary antibodies (suppl. table S1) overnight at 4 °C. Secondary antibodies (suppl. table S1) were added for one hour RT in the dark. Pictures were taken using the EVOS M7000 Imaging System (RRID: SCR_025070).

### Immunofluorescence

FFPE tumor sections were deparaffinized in xylene and dehydrated with increasing dilutions of ethanol. Antigen retrieval was performed in 10 mM citric acid monohydrate buffer at pH 6.5. Non-specific binding was blocked using 20% human AB serum for 30 min at RT. Primary antibodies (suppl. table S1) were applied overnight at 4 °C. Secondary antibodies (suppl. table S1) were incubated for one hour at RT followed by Hoechst staining (1:2000 in PBS) for 8 min. Slides were mounted with Prolong Gold Antifade reagent (ThermoFisher).

### LC-MS/MS

Steroid profiles of ACC tissues were determined using liquid chromatography tandem mass spectrometry (LC-MS/MS) as previously described^22^.

### Western blot

Cells were lysed in RIPA buffer (Sigma) supplemented with 1% Protease Inhibitor (Sigma), 1% Phosphatase B Inhibitor, and 2% Phosphatase C Inhibitor (Santa Cruz). Protein concentrations were quantified using the Pierce™ BCA Protein Assay Kit. Absorbance values were measured at the BMG Labtech FLUOstar Omega (RRID: SCR_025024) microplate reader at 562 nm. Protein was loaded onto a 4–20% Mini-PROTEAN^®^ TGX™ Precast Protein Gel (Bio-Rad) and separated by SDS-PAGE. Proteins were transferred by semi-dry blot onto a nitrocellulose membrane (Cytiva) that was subsequently blocked in 5% skimmed milk in TBS-Tween at RT for 1 h. Primary antibodies (suppl. Table 1) were diluted in 5% skimmed milk in TBS-Tween and incubated overnight at 4 °C. Membranes were washed and incubated at RT for one hour with horseradish-peroxidase (HRP)-labeled secondary antibodies. Immunoblots were developed and visualized using Clarity™ Western ECL Substrate at the Bio-Rad Chemidoc XRS Gel Imaging System (RRID: SCR_019690) with Image Lab Software (RRID: SCR_014210). GAPDH expression was used as a loading control.

### Nanostring nCounter gene expression analysis

Gene expression analysis was performed on RNA isolated either from *in vitro* differentiated macrophages or from FFPE ACC tissue sections. RNA was extracted using the Maxwell RSC simplyRNA Tissue Kit (Promega) or the AllPrep DNA/RNA FFPE Kit (Qiagen), respectively. RNA quantity and quality were assessed with a NanoDrop2000 spectrophotometer (Thermo Fisher Scientific), and samples meeting predefined quality criteria were further analyzed for gene expression quantification with the NanoString nCounter Analysis System (RRID: SCR_021712). Customized panels (NanoString Technologies (RRID: SCR_023912)) consisting of immune-related and glucocorticoid-related genes plus eleven housekeeping genes were used according to the manufacturer´s instructions. Analysis was performed by nSolver Analysis Software (RRID: SCR_003420). Samples passing imaging quality control thresholds (fields of view read > 75%, binding density of 0.05–2.25, positive control linearity > 0.95, and positive control detection limit > 2) were normalized using technical controls and housekeeping genes, based on positive control normalization with the geometric mean as the normalization factor. For comparisons, a one-tailed Student’s t-test p-value and a false discovery rate (FDR) were calculated by the Benjamini–Yekutieli method. Genes were ranked by fold change (FC) and FDR, with thresholds set at FC ≥ 2 and FDR < 0.1.

### Phagocytosis assay

JIL-2266 were stained with CFSE (BioTracker 488 Green CFSE Cell Proliferation Kit, Sigma, Cat# SCT110) and seeded in 12 well plates at a density of 1,5*10^5 cells/well. After 24 h of incubation at 37 °C, cells were treated with RSL3 for 2 h. Medium was discarded, and the wells were washed with PBS twice. MФ were added to the cultured early ferroptotic ACC cells at a ratio of 7:1 tumor cell: MФ overnight. This ratio was chosen to reflect the tumor-to-macrophage proportion observed in the ACC tumor microenvironment (~ 15% macrophages). For inhibition studies, MФ were pre-incubated with the selective MerTK inhibitor UNC1062 (100 nM; MCE: Cat# HY-117548), anti-Gas6 antibody (20 µg/ml; R&D Systems, Cat# AB885, RRID: AB_354376), a goat isotype IgG control (20 µg/ml; Thermo Fisher Scientific, Cat# 02–6202, RRID: AB_2532946), purified C1q (Merck; Cat# C1740) or anti-C1QA antibody (LS-Bio; Cat# LS‑C100911; RRID: AB_2067267) for 30 min before they were added to early ferroptotic tumor cells. Flow cytometry was used to monitor ferroptotic cell death by Annexin V/propidium iodide (PI) staining (BioLegend, Cat# 640932) and to quantify percentages of CFSE+ CD64 + macrophages. Therefore, co-cultured ferroptotic ACC cells and macrophages were trypsinized and washed with PBS. Anti-CD64 antibody was diluted 1:100 in PBS and incubated with cells for 30 min at RT. Cells were washed with PBS and incubated with the respective secondary antibody for 30 min at RT, protected from light. Cells were washed and resuspended in 200 µl FACS buffer. For additional intracellular C1QA staining, cells were fixed and permeabilized after staining with the primary anti-CD64 antibody using the FoxP3/Transcription Factor Staining Buffer Set (eBioscience; Cat# 00–5523-00), according to the manufacturer’s instructions. Anti-C1QA antibody was diluted 1:20 in permeabilization buffer and incubated with the cells for 30 min at 4 °C. Cells were then washed, and both secondary antibodies were added simultaneously and incubated for 30 min at room temperature in the dark. The percentages of CFSE+ CD64 + cells were determined using a FACS-Fortessa at the Ludwig Maximilian University Hospital Munich Flow Cytometry Core Facility (RRID: SCR_026395). The flow cytometry results were analyzed using FlowJo™ v10.8 Software (BD Life Sciences) (RRID: SCR_008520)^23^.

### ELISA

CXCL9 concentrations in cell culture supernatants and plasma were measured by ELISA (CXCL9/MIG: Invitrogen, Cat# 900-K87K). If not stated otherwise, concentrations were normalized to total protein levels isolated as stated above.

### Survival and co-expression analysis

To assess RNA co-expression and the prognostic significance of gene expression levels, we performed survival analysis using clinical and RNAseq data from The Cancer Genome Atlas (TCGA) project and a local ACC biobank (NCT MASTER). For survival analyses, patients were stratified into high- and low-expression groups based on the median expression level of each gene of interest. Survival curves were generated using Kaplan-Meier estimates and exported from the cBioPortal platform (TCGA). Co-expression plots were either exported from cBioportal or generated using R Project for Statistical Computing (RRID: SCR_001905) version R/4.0.0 (NCT MASTER). Differences in survival between groups were assessed using the log-rank test. For the use of data from a public database, an approval exemption has been obtained from the LMUs´ ethics committee.

### Gene set enrichment analysis

For pathway and functional enrichment analysis, we employed Gene Set Enrichment Analysis (GSEA) using the WebGestalt: WEB-based GEne SeT AnaLysis Toolkit (RRID: SCR_006786). Differentially expressed genes identified from our RNA dataset were input into WebGestalt to assess their association with known biological pathways, gene ontology (GO) categories, and functional gene sets.

### Mice

All animal procedures were approved by the Institutional Animal Care and Use Committee of Boston Children’s Hospital, and all methods were performed in accordance with relevant guidelines and regulations. Animal experiments were reported in accordance with the ARRIVE guidelines. Wild-type male C57BL/6J mice (Jackson Laboratory, #000664) were used. The BCH-ACC3 cell line was established from a 538 mg primary glucocorticoid-producing ACC tumor harvested from a male BPCre mouse with lung metastases^24^, as previously described^25^. The primary tumor was dissociated using the Tumor Dissociation Kit, mouse (Miltenyi Biotec, 130-096-730), and serially transplanted subcutaneously into C57BL/6J mice over six months. Subsequently, cells were cultured *in vitro* in Advanced DMEM/F12 medium (Thermo Fisher, Cat# 12634028) supplemented with 2% Nu-Serum (Thermo Fisher CB-5100), 1% ITS (Corning, Cat# 354350), 2 mM L-glutamine (Thermo Fisher, Cat# 35050061), and 1% penicillin/streptomycin (Thermo Fisher, Cat# 15140122).

### Animal experiments

BCH-ACC3 cells (3 × 10^6^) were diluted in 100 µL PBS/Matrigel (1:1) and injected subcutaneously in the right flank of 8–9-week-old male C57BL/6J (Jackson). Mice received isoflurane inhalation anesthesia (induction at 3–5% and maintenance at 1–3% in oxygen) delivered via a precision vaporizer. Depth of anesthesia was assessed by monitoring respiratory rate and loss of pedal reflex. Tumors were measured by electronic caliper on day 6 post-implant, and volume was calculated using the formula volume=length x (width)^2^/2. Mice were randomized to treatment groups, and treatment was commenced on the same day. Mifepristone (diluted in 0.9% Saline with 10% ethanol and 6.25% Tween 80) was administered intraperitoneally (i.p.) at a dose of 40 mg/kg once daily from day 6 onwards. An immune checkpoint inhibitor (ICI) cocktail of anti-PD1 antibody (200 µg per mouse) and anti-CTLA4 antibody (200 µg per mouse) was administered i.p. on days 6, 9, and 12. Mifepristone was purchased from Sigma (Cat# M8046). Anti-PD1 (Cat# BE0273) and anti-CTLA4 (Cat# BE0164) antibodies were purchased from Bio X Cell. Mice were euthanized and tumors were collected on day 15, weighed, and used for immunoprofiling by flow cytometry and IHC. Mice were euthanized using compressed 100% CO₂ gas delivered from a regulated cylinder, in accordance with the AVMA Guidelines for the Euthanasia of Animals (2020) and NIH’s *Guidelines for Euthanasia of Rodents Using Carbon Dioxide*. Animals were placed in an empty euthanasia chamber (not pre-filled), and CO₂ was introduced at a controlled fill rate of 30–70% of the chamber volume per minute, ensuring gradual displacement of room air and minimizing distress. Animals were continuously observed throughout the procedure. CO₂ flow was maintained for at least 1 min after respiratory arrest, and death was confirmed by lack of respiration and fixed, dilated pupils. All procedures were performed by trained personnel using properly calibrated equipment.

### Tumor immunoprofiling of mice by flow cytometry

Tumor masses were mechanically diced, and a single-cell suspension was prepared using the mouse tumor dissociation kit (Miltenyi Biotec) according to the manufacturer’s instructions. After filtering through a 70 μm filter, cells were washed and stained with fluorochrome-conjugated monoclonal antibodies. Samples were acquired using BD LSRII flow cytometer. The flow cytometry results were analyzed using FlowJo™ v10.8 Software (BD Life Sciences)^23^. Antibodies used for flow cytometry: mouse CD8a (Biolegend, RMG1-1), mouse CD3 (Biolegend, 17A2), mouse CD4 (Biolegend, GK1.5), CD11c (Biolegend, N418), mouse F4/80 (Biolegend, BM8), mouse CD11b (Biolegend, M1/70).

### Multi-parametric flow cytometry analysis of PBMCs

Patient-derived PBMCs were isolated on the day of blood collection and frozen in DMEM high glucose, 10% FBS, and 10% DMSO at 80 °C until use. For flow cytometry analysis, PBMCs were stained for cell-surface markers CD3, CD4, CD8, and CXCR3. Briefly, thawed PBMCs were incubated with fixable viability dye (ThermoFisher, Cat# 65–0865-14) to discriminate live and dead cells. Following viability staining, cells were washed and incubated with Fc receptor blocking reagent (Biolegend, 422302) to prevent nonspecific antibody binding. After blocking, cells were stained with fluorochrome-conjugated antibodies against CD3, CD4, CD8, and CXCR3 (suppl. Table 1) at indicated concentrations for 30 min at 4 °C in the dark in cell staining buffer (Biolegend, 420201). Flow cytometry was conducted using the FACS-Fortessa at the Ludwig Maximilian University Hospital Munich Flow Cytometry Core Facility (RRID: SCR_026395). The flow cytometry results were analyzed using FlowJo™ v10.8 Software (BD Life Sciences) (RRID: SCR_008520)^23^.

### Statistical analysis

Unless otherwise stated, three independent replicates were performed per experiment. For experiments using human MΦ, each biological replicate equals a different biological blood donor. Statistical analyses were performed using GraphPad Prism (RRID: SCR_002798) version 9.0. If not stated otherwise, biological replicates are shown in Mean +/- SD, and the statistical analyses were performed using a two-tailed Student’s t test for paired comparisons or one-way analysis of variance (ANOVA) for multiple comparisons. *p < 0.05, **p < 0.01, ***p < 0.001, ****p < 0.0001, ns = not significant.

## Results

### Macrophages in ACC are frequent irrespective of tumoral glucocorticoid excess and exhibit a CD68 + CD163+ phenotype

**Fig. 1:**
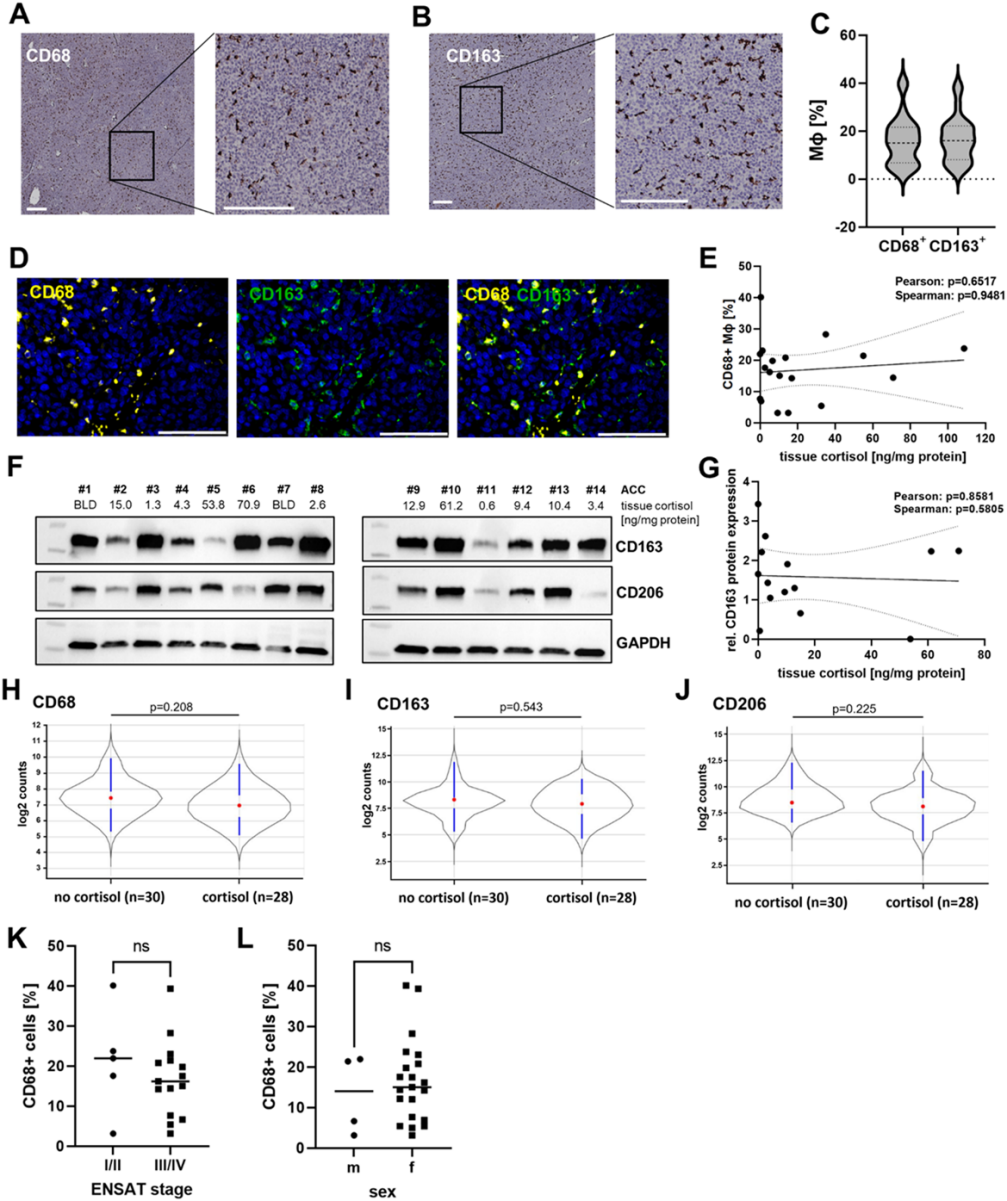
Macrophages in ACC. (**A**,** B**) Immunohistochemistry (IHC) for macrophage markers was performed in tumor tissues from 25 ACC patients. Representative images of (A) CD68 staining and (B) CD163 staining are shown. Scale bar: 200 μm. (**C**) The percentage of IHC-positive cells was quantified using positive cell detection (QuPath). Median percentage of positive cells: 15% (CD68+) and 16% (CD163+), dashed lines represent medians, dotted lines represent upper and lower quartiles. *N* = 25. (**D**) Co-Immunofluorescence of CD68 and CD163 was performed in tumor samples from ACC patients. A representative image is shown. Scale bar: 100 μm. (**E**) The percentage of CD68 + cells was plotted against intra-tumoral cortisol levels. Cortisol was quantified by LC-MS/MS. *N* = 18. (**F**) Immunoblots showing CD163 and CD206 expression in protein lysates of ACC tumors. Tissue cortisol concentrations were determined by LC-MS/MS. BLD=below limit of detection. *N* = 14. (**G**) Relative CD163 protein expression in whole tumor lysates of ACC tumors was quantified using ImageJ and correlated with intra-tumoral cortisol levels. (**H-J**) Violin plots displaying the total tumoral expression of (H) *CD68*, (I) *CD163* and (J) *CD206* in a total of 58 ACC tumors, stratified by clinical cortisol excess; Red dots represent medians, blue lines connect lower adjusted value and lower quartile and upper adjusted value and upper quartile, respectively. FDR-adjusted p-values were calculated using the Benjamin&Yekutieli correction and the Nanostring nSolver software. **(K**) Correlation of CD68 + cell percentages and ENSAT tumor stage. *N* = 20. Differences were compared using Mann-Whitney-U-test. (**L**) Correlation of CD68 + cell percentages and sex. *N* = 25. Differences were compared using Mann-Whitney-U-test; m= male, f=female.

Using chromogenic immunohistochemistry (IHC), we investigated the proportion of TAMs in tissue samples from 25 patients with ACC. We observed a heterogeneous but generally abundant population of CD68 + cells, reflecting macrophage infiltration (Fig. 1A). Furthermore, CD163 + cells, indicative of an M2-like macrophage phenotype, were consistently present in all ACC tissues examined (Fig. 1B). Intra-tumoral TAM counts varied from 3.1% to 40.1% (median = 15.0%) for CD68 + cells and 3.0% to 37.6% (median = 16.0%) for CD163 + cells (Fig. 1C). Moreover, immunofluorescence microscopy revealed the near-total co-localization of CD68 and CD163 in ACC tissue samples (Fig. 1D). Tissue cortisol levels measured using LC-MS/MS did not correlate with the total TAM population assessed by IHC staining (Spearman: p = 0.95; Fig. 1E). Likewise, we did not observe a significant correlation between intra-tumoral cortisol concentrations and the total tumoral expression of the ‘M2-like’ macrophage marker CD163 and CD206, as determined by immunoblotting of the same tumor lysates used for steroid hormone quantification to minimize the impact of intra-tumoral heterogeneity (Spearman: *p* = 0.58; Fig. 1F, G). In a separate cohort of 58 ACC patients stratified by clinical hormone status, tumoral RNA expression of *CD68*, *CD163*, and *CD206* did not differ between patients with or without cortisol excess (Fig. 1H-J). Moreover, when tumors were categorized as inactive, androgen-overproducing, GC-overproducing, or combined androgen and GC excess, no significant differences in the expression of these markers were observed across the four groups (Fig. S1A-C). Additionally, in our cohort, the number of infiltrating macrophages determined by IHC staining did not differ by tumor stage (Fig. 1K) and sex (Fig. 1L). Altogether, these findings suggest that MΦ are a heterogeneous but prevalent immune cell population in ACC, predominantly exhibiting a CD68 + CD163+ phenotype. The quantity and polarization of these macrophages appeared to be independent of intra-tumoral cortisol levels, ENSAT stage, or sex.

### Glucocorticoids induce MΦ polarization towards a C1Q+ CD163 + phenotype

**Fig. 2:**
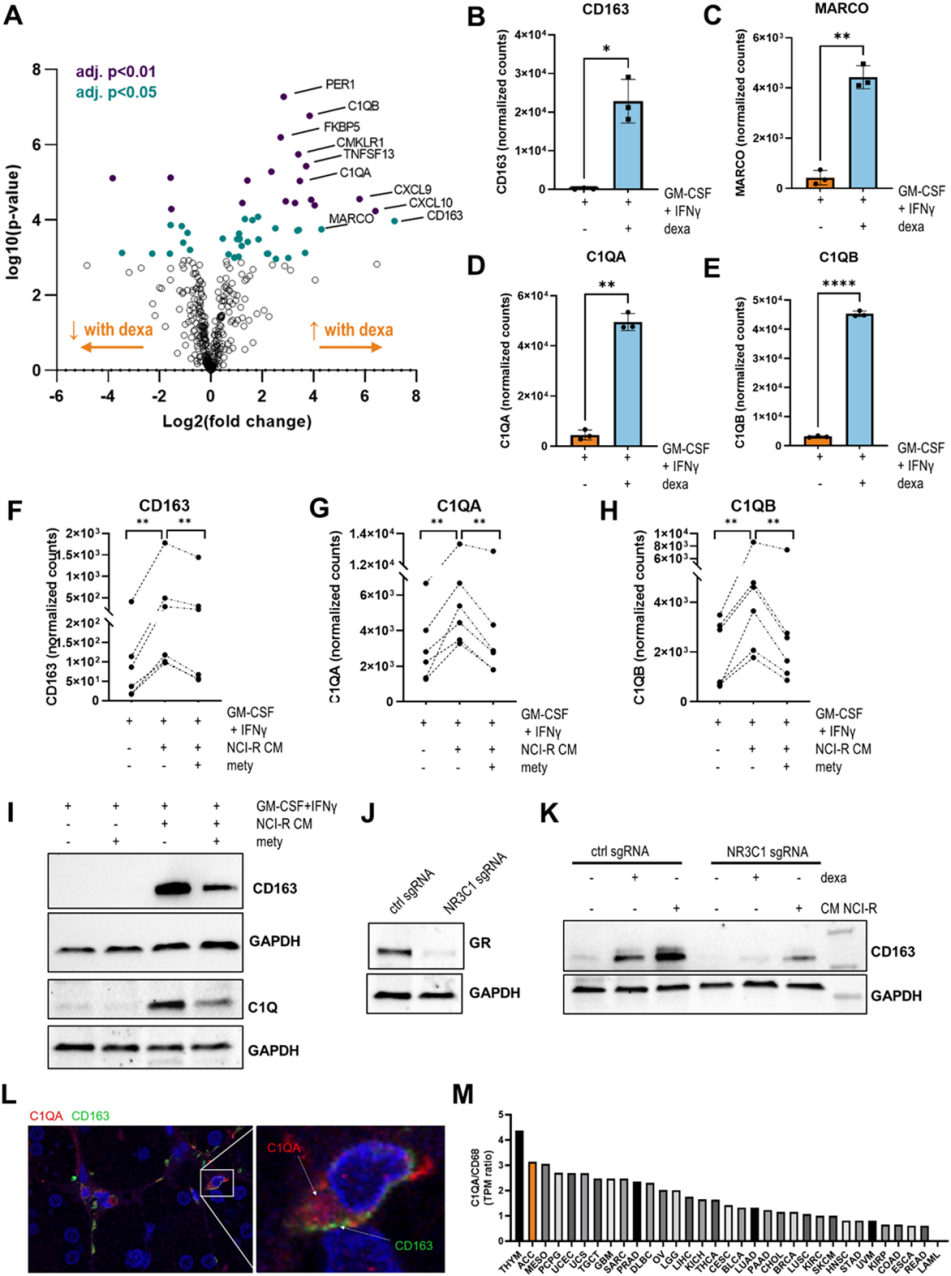
Glucocorticoid-induced MΦ polarization. (**A**) Volcano plot showing differentially expressed genes (DEGs) in MΦ exposed to GM-CSF+IFNγ vs. GM-CSF+IFNγ + dexamethasone (100 nM; added from day 1 onwards). Analysis of RNA data was performed with Nanostring nCounter. FDR-adjusted p-values were calculated using the Benjamin&Yekutieli correction and the Nanostring nSolver software. *N* = 3 biological replicates from independent healthy blood donors. (**B-E**) Expression of (B) *CD163*, (C) *MARCO*, (D) *C1QA* and (E) *C1QB* in MΦ exposed to GM-CSF+IFNγ vs. GM-CSF+IFNγ + dexamethasone (100 nM). *N* = 3 biological replicates from independent healthy blood donors are shown in mean +/- SD. Differences were compared using paired t-test. (**F-I**) *CD163*, (G) *C1QA*, (H) *C1QB* RNA and (I) CD163 and C1Q protein expression in MΦ exposed to GM-CSF+IFNγ and CM of NCI-H295R cells cultured in the presence or absence of the steroidogenesis inhibitor metyrapone (10 µM) vs. controls. *N* = 3 biological replicates from independent healthy blood donors. Differences were compared using paired t-test. (**J**) SgRNA knockdown of the glucocorticoid receptor (GR; gene: *NR3C1*) in THP-1 MΦ. (K) CD163 protein expression in THP-1 *NR3C1* KO and control cells in response to dexamethasone or NCI-H295R CM exposure. (**L**) Co-Immunofluorescence of C1QA and CD163 was performed in adrenocortical tissue. One representative image is shown. **(M**) Analysis of *C1QA*/*CD68* gene expression ratio across different cancer entities. Data was obtained from TCGA. NCI-R = NCI-H295R; mety=metyrapone; CM=conditioned media; BLCA: Bladder Urothelial Carcinoma, BRCA: Breast Invasive Carcinoma, CESC: Cervical Squamous Cell Carcinoma and Endocervical Adenocarcinoma, CHOL: Cholangiocarcinoma, COAD: Colon Adenocarcinoma, DLBC: Diffuse Large B-cell Lymphoma, ESCA: Esophageal Carcinoma, GBM: Glioblastoma Multiforme, HNSC: Head and Neck Squamous Cell Carcinoma, KICH: Kidney Chromophobe, KIRC: Kidney Renal Clear Cell Carcinoma, KIRP: Kidney Renal Papillary Cell Carcinoma, LAML: Acute Myeloid Leukemia, LGG: Brain Lower Grade Glioma, LIHC: Liver Hepatocellular Carcinoma, LUAD: Lung Adenocarcinoma, LUSC: Lung Squamous Cell Carcinoma, MESO: Mesothelioma, OV: Ovarian Serous Cystadenocarcinoma, PAAD: Pancreatic Adenocarcinoma, PCPG: Pheochromocytoma and Paraganglioma, PRAD: Prostate Adenocarcinoma, READ: Rectum Adenocarcinoma, SARC: Sarcoma, SKCM: Skin Cutaneous Melanoma, STAD: Stomach Adenocarcinoma, TGCT: Testicular Germ Cell Tumors, THCA: Thyroid Carcinoma, THYM: Thymoma, UCEC: Uterine Corpus Endometrial Carcinoma, UCS: Uterine Carcinosarcoma, UVM: Uveal Melanoma.

Although the amount of GCs produced by the tumor did not influence the number of infiltrating macrophages (Fig. 1E-J), we hypothesized that the generally high prevalence of steroid hormones in adrenal tumors - both in patients with and without clinical peripheral hormone excess - may affect their exact polarization state. Therefore, we further investigated the impact of GCs on macrophage polarization *in vitro* by determining the gene expression profile of macrophages exposed to the synthetic glucocorticoid dexamethasone using a custom Nanostring nCounter panel. To better assess gene expression changes by dexamethasone, M1-polarized macrophages (polarized in the presence of GM-CSF + IFNγ) were used as controls, while a GC-derived macrophage phenotype was induced by simultaneous treatment with M1-polarizing factors and dexamethasone. In addition, this setup circumvented the use of M-CSF, which was shown to already induce M2 polarization of macrophages (Fig. S2A). Co-exposure of primary monocytes to dexamethasone and GM-CSF + IFNγ resulted in a strong upregulation of the macrophage marker *CD163* and *MARCO* compared to GM-CSF + IFNγ monotreatment (Fig. 2A-C). Moreover, genes encoding subunits of the complement component C1q, in particular *C1QA* and *C1QB*, were among the most discriminatively upregulated transcripts in comparison to M1-polarized macrophages (Fig. 2A, D, E). Flow cytometry analysis of C1QA and CD163 expression in macrophages confirmed the presence of a C1Q+ CD163 + phenotype following dexamethasone treatment (Fig. S2B, C). To generate TAMs that more closely resemble tumor-induced macrophage polarization, we next used conditioned medium (CM; 33% v/v) from the steroidogenic ACC cell line NCI-H295R to polarize macrophages *in vitro*. We observed clear morphologic changes (Fig. S2D) alongside significantly elevated expression of *CD163*, *C1QA*, and *C1QB* in macrophages cultured in the presence of NCI-H295R CM. Remarkably, this increase was abrogated when ACC cells were cultured in medium containing the steroidogenesis inhibitor metyrapone prior CM collection (Fig. 2F-I, Fig. S2E). In macrophages derived from THP-1 monocytes, knockout of the glucocorticoid receptor (GR; gene: *NR3C1*) efficiently attenuated CD163 expression upon treatment with dexamethasone or NCI-H295R CM, further supporting that macrophage polarization following exposure to CM of ACC cells was primarily driven by a GC-GR dependent mechanism (Fig. 2J, K). However, in this cell line, neither C1Q expression nor C1q secretion was detected in either activation state. Supporting the presence of a C1Q+ CD163+ MΦ phenotype in ACC tissue, co-immunofluorescence analysis revealed cytosolic staining of C1QA in cells that were also positive for CD163 (Fig. 2L). Moreover, *C1QA* gene expression was among the most significantly upregulated genes in ACC tumors with high CD163 expression levels (Fig. S2F). A pan-cancer analysis of TCGA data demonstrated that the *C1QA/CD68* gene expression ratio was notably elevated in ACC tumor samples compared to other tumor types (Fig. 2M). Within our ACC cohort, we identified a trend towards a higher *C1QA/CD68* gene expression ratio (*p* = 0.0963) in samples with high expression of the steroidogenic enzyme CYP11B1, suggesting that the extent of GC synthesis may not further enhance C1Q+ macrophage polarization (Fig S2G). Notably, GC production was recently detected also in several non-adrenal tumor types, produced by recycling of inactive metabolites via 11β-hydroxysteroid dehydrogenase type 1 (11β-HSD1, encoded by HSD11B1)^3^. Interrogation of TCGA data identified a significantly increased *C1QA/CD68* gene expression ratio in tumor samples with high *HSD11B1* expression across these cancer types (Fig. S2H), further supporting a link between local GC production and C1Q+ macrophages in diverse tumor microenvironments.

### GC-induced MΦ are highly efficient in C1q-mediated phagocytosis and correlate with better survival in ACC

**Fig. 3:**
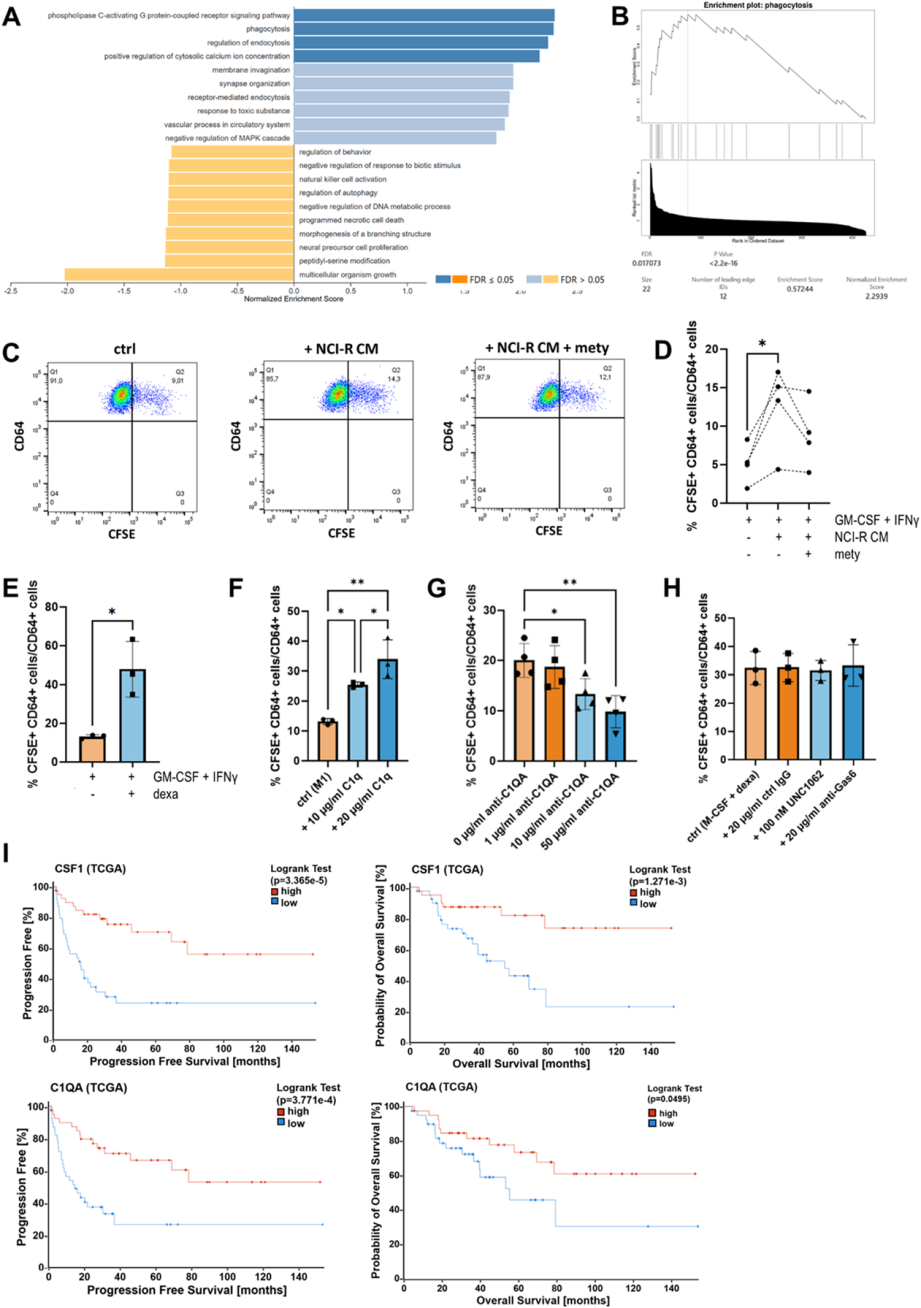
GC-induced MΦ are highly efficient in C1q-mediated phagocytosis. (**A**) Gene set enrichment analysis (GSEA) of DEGs in MΦ exposed to CM of steroidogenic NCI-H295R cells in comparison to controls. (**B**) Visualization of the enrichment results on phagocytosis (*p* < 2.2e-16; FDR: 0.011718). (**C**) Representative flow cytometry plots and (**D**) quantification of phagocytosis by MΦ exposed to CM of steroidogenic NCI-H295R cells or control. CD64 + CFSE+ double-positive cells were considered phagocytic macrophages. Groups were compared using one-way ANOVA with Tukey’s multiple comparisons test. *N* = 4 biological replicates from independent healthy blood donors (**E**) Percentages of phagocytic MΦ exposed to dexamethasone (100 nM) or control. *N* = 3 biological replicates from independent healthy blood donors are shown in mean +/- SD. Differences were compared using unpaired t-test. (**F**) Phagocytosis of M1-polarized MΦ after exposure to 10 µg/ml or 20 µg/ml purified human C1q. *N* = 3 biological replicates from independent healthy blood donors are shown in mean +/- SD. Groups were compared using one-way ANOVA with Tukey’s multiple comparisons test. (**G**) Phagocytosis of dexamethasone (100 nM) -polarized MΦ after exposure to 1 µg/ml, 10 µg/ml or 50 µg/ml anti-C1QA antibody. *N* = 3 biological replicates from independent healthy blood donors are shown in mean +/- SD. Groups were compared using one-way ANOVA with Tukey’s multiple comparisons test. (**H**) Phagocytosis of dexamethasone (100 nM)-polarized MΦ after exposure to the MerTK inhibitor UNC1062 (100 nM), anti-Gas6 antibody (20 µg/ml) or goat control IgG (20 µg/ml). *N* = 3 biological replicates from independent healthy blood donors are shown in mean +/- SD. Groups were compared using one-way ANOVA with Tukey’s multiple comparisons test. (**I**) Kaplan-Meier plots of PFS and OS according to tumoral *CSF1* and *C1QA* expression. Higher gene expression was significantly associated with favorable PFS and OS. RNA expression and survival data was extracted from the TCGA dataset (*n* = 76). NCI-R = NCI-H295R; mety=metyrapone.

To evaluate the functional relevance of the GC-induced C1Q+ macrophage phenotype, we next conducted a gene set enrichment analysis (GSEA) of RNA data obtained from the Nanostring nCounter analysis. This analysis revealed a statistically significant enrichment (*p* < 2.2e-16; FDR: 0.017) of gene sets associated with phagocytosis in macrophages polarized with CM of the steroidogenic ACC cell line NCI-H295R (Fig. 3A, B). Following this finding, we next co-cultured GC-induced macrophages with ACC cells and assessed their phagocytic capacity. As we have previously shown that ferroptosis is a prominent mode of cell death in adrenocortical cells^26^, tumor cell death was initiated by treatment with the ferroptosis inducer RSL3. Cell death was monitored by Annexin V/PI staining, which showed a substantial increase in phosphatidylserine (PS) exposure in early ferroptotic cells (Fig. S3A, B). Consistent with the GSEA results, the co-culture with NCI-H295R CM-exposed macrophages showed an increased percentage of phagocytic macrophages (% [CFSE+ CD64 + cells/total CD64 + cells]) compared to control-treated macrophages (Fig. 3C, D; gating strategy shown in Fig. S3C). Similarly, macrophages polarized with dexamethasone demonstrated significantly increased phagocytic capacity relative to M1-polarized macrophages (Fig. 3E). Additionally, these macrophages exhibited a notable, though not statistically significant, upregulation of genes encoding MHC class II molecules, suggesting adequate antigen presentation (Fig. S3D, E). Given that secreted C1q has been shown to play a key role in the clearance of target cells by recognizing PS on their surface, thereby marking them for phagocytosis^27,28^, we next investigated whether adding serum-derived C1q to M1-polarized macrophages would enhance their phagocytic capacity. Indeed, exposure of co-cultured ACC cells and macrophages to C1q resulted in a dose-dependent increase in CFSE+ macrophages (Fig. 3F). Conversely, the addition of an anti-C1QA antibody to dexamethasone-polarized macrophages significantly reduced phagocytic activity, further supporting a role of secreted C1q in mediating phagocytosis by GC-polarized macrophages. (Fig. 3G). Additionally, C1QA staining in these macrophages revealed higher expression in CFSE+ phagocytic macrophages compared with CFSE- macrophages (Fig. S3F, G). We also observed that GCs induce MerTK expression in macrophages (Fig. S3H), an alternative mediator of phagocytosis via the MerTK/Gas6/PS axis. Intriguingly, phagocytosis by C1Q+ macrophages was not affected by treatment with the selective MerTK antagonist UNC1062 or by administration of a neutralizing antibody against its ligand Gas6 (Fig. 3H). Supporting an essential role for phagocytic macrophages in ACC, we found that expression of *C1QA* and the macrophage growth factor *CSF1* positively correlated with improved overall and progression-free survival in the TCGA dataset (Fig. 3I). In conclusion, these results demonstrate that GCs induce a highly phagocytic macrophages phenotype, primarily mediated by increased expression and secretion of the complement component C1q, rather than through the MerTK pathway. The presence of these macrophages may be associated with improved prognosis in patients with ACC.

### IFNγ treatment of C1Q+ MΦ strongly induces CXCL9 expression

**Fig. 4:**
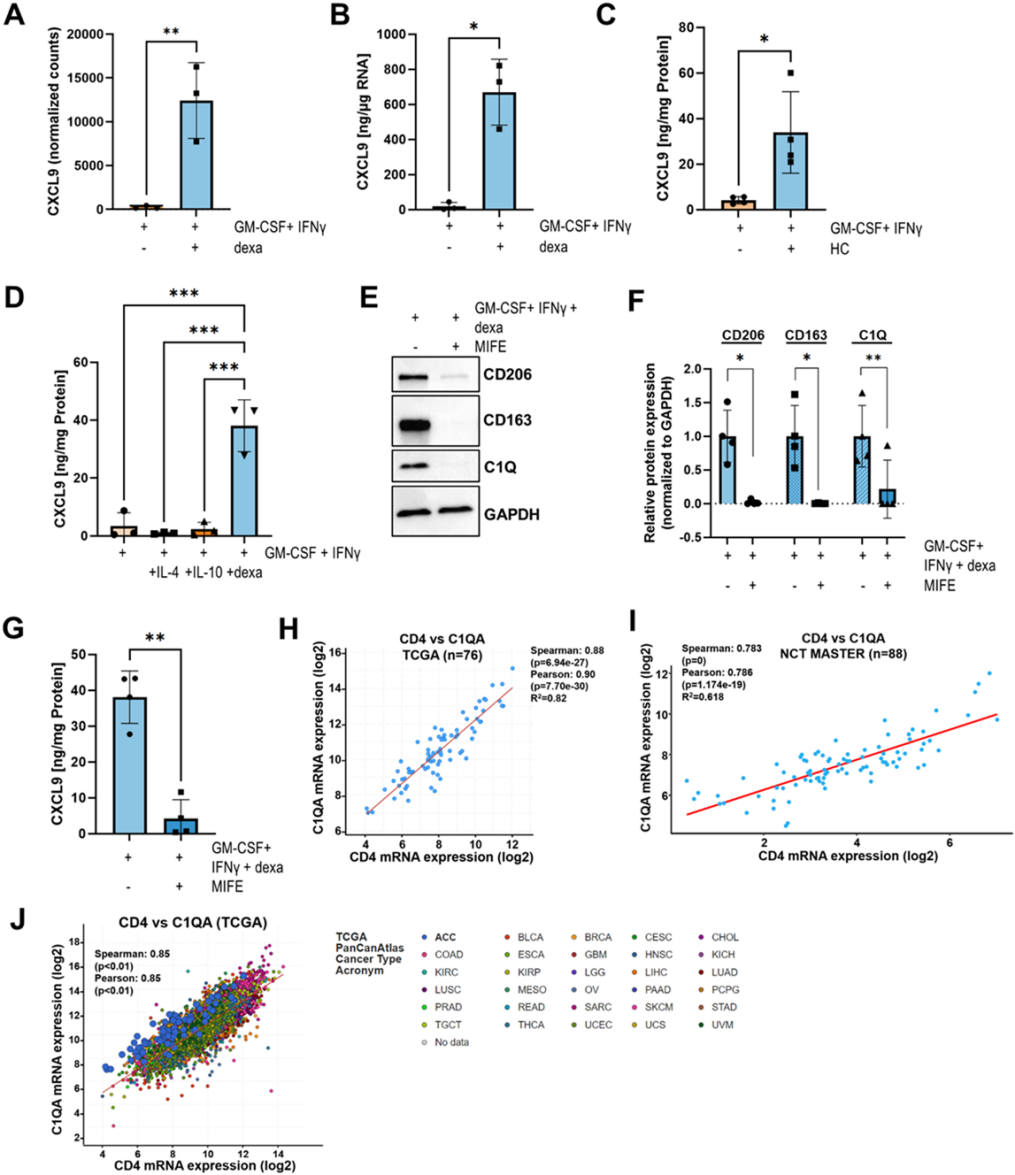
IFNγ stimulates CXCL9 release from C1Q⁺ macrophages. (**A**) *CXCL9* RNA expression in MΦ exposed to GM-CSF+IFNγ vs. GM-CSF+IFNγ + dexamethasone (100 nM). *N* = 3 biological replicates from independent healthy blood donors are shown in mean +/- SD. Statistical analysis was performed using paired t-test. (**B**) CXCL9 secreted from MΦ exposed to GM-CSF+IFNγ vs. GM-CSF+IFNγ and dexamethasone (100 nM). CXCL9 was normalized to total RNA isolated for gene expression analysis. *N* = 3 biological replicates from independent healthy blood donors are shown in mean +/- SD. Differences were compared using paired t-test. (**C**) CXCL9 secreted from MΦ exposed to GM-CSF+IFNγ vs. GM-CSF+IFNγ and hydrocortisone (HC; 500 ng/ml). *N* = 4 biological replicates from independent healthy blood donors are shown in mean +/- SD. Statistical comparisons were performed using paired t-test. (**D**) CXCL9 secreted from MΦ exposed to GM-CSF+IFNγ vs. GM-CSF+IFNγ and IL-4 (20 ng/ml), IL-10 (50 ng/ml) or dexamethasone (100 nM). *N* = 3 biological replicates from independent healthy blood donors are shown in mean +/- SD. Groups were compared using one-way ANOVA with Tukey’s multiple comparisons test. (**E**) CD163, CD206 and C1Q protein expression in MΦ exposed to GM-CSF+IFNγ vs. GM-CSF+IFNγ and mifepristone (10 µM). A representative Western blot is shown. (**F**) Quantified CD163, CD206 and C1Q protein expression in MΦ exposed to GM-CSF+IFNγ vs. GM-CSF+IFNγ and mifepristone (10 µM). *N* = 4 biological replicates from independent healthy blood donors are shown in mean +/- SD. Differences were compared using paired t-test. (**G**) CXCL9 secreted from MΦ exposed to GM-CSF+IFNγ vs. GM-CSF+IFNγ and mifepristone (10 µM). *N* = 4 biological replicates from independent healthy blood donors are shown in mean +/- SD. Differences were compared using paired t-test. (**H-I**) Correlation of *C1QA* and *CD4* expression in ACC tumors within the (H) TCGA dataset (*n* = 76) or (I) NCT MASTER dataset (*n* = 88). (**J**) Pan-cancer correlation of *C1QA* and *CD4* gene expression. Data was obtained from TCGA. GC=glucocorticoid; PFS=progression free survival; dexa=dexamethasone; HC=hydrocortisone.

Upon IFNγ stimulation, the T cell chemoattractants *CXCL9* and *CXCL10* were among the most significantly upregulated genes in macrophages exposed to dexamethasone compared with M1-polarized macrophages (Figs. 2A and 4A). Consistently, CXCL9 concentrations were also significantly increased in the cell culture supernatant, as quantified by ELISA (Fig. 4B, absolute CXCL9 concentrations shown in Fig. S4A). Likewise, exposure to hydrocortisone strongly increased CXCL9 secretion by macrophages upon treatment with IFNγ (Fig. 4C, Fig. S4B). Yet, a full establishment of the GC-induced C1Q+ macrophage phenotype appeared to be critical in this context, as simultaneous application of dexamethasone and IFN-γ reduced the expression of C1Q and the increase in CXCL9 production (Fig. S4C-E). Accordingly, macrophages exposed to dexamethasone after IFNγ treatment did not produce higher amounts of CXCL9 compared to M1-polarized macrophages (Fig. S4F, G). Moreover, increased CXCL9 production was exclusive to GC-polarized macrophages and was not observed in macrophages polarized by IL-4 or IL-10 prior to IFNγ stimulation (Fig. 4D, Fig. S4H). Concurrent exposure of macrophages to dexamethasone and the GR antagonist mifepristone blunted both the macrophage phenotype and IFN-γ-induced secretion of CXCL9 (Fig. 4E-G, Fig. S4I). As CXCL9 and CXCL10 are highly potent T cell attractants through binding to their receptor CXCR3, predominantly expressed on T cells^29^, we next analyzed the connection between this macrophage phenotype and T cell infiltration in ACC. In two independent datasets, the complement component *C1QA* strongly correlated with *CD3D*, *CD8A*, and most significantly with *CD4* gene expression (Fig. 4H, I; Fig. S4J-M). Moreover, a pan-cancer analysis of TCGA data revealed this correlation to be true across several cancer entities (Fig. 4J).

### In mice, immunotherapy-driven tumoral CD4 + T cell infiltration is significantly hampered by concurrent treatment with mifepristone

**Fig. 5:**
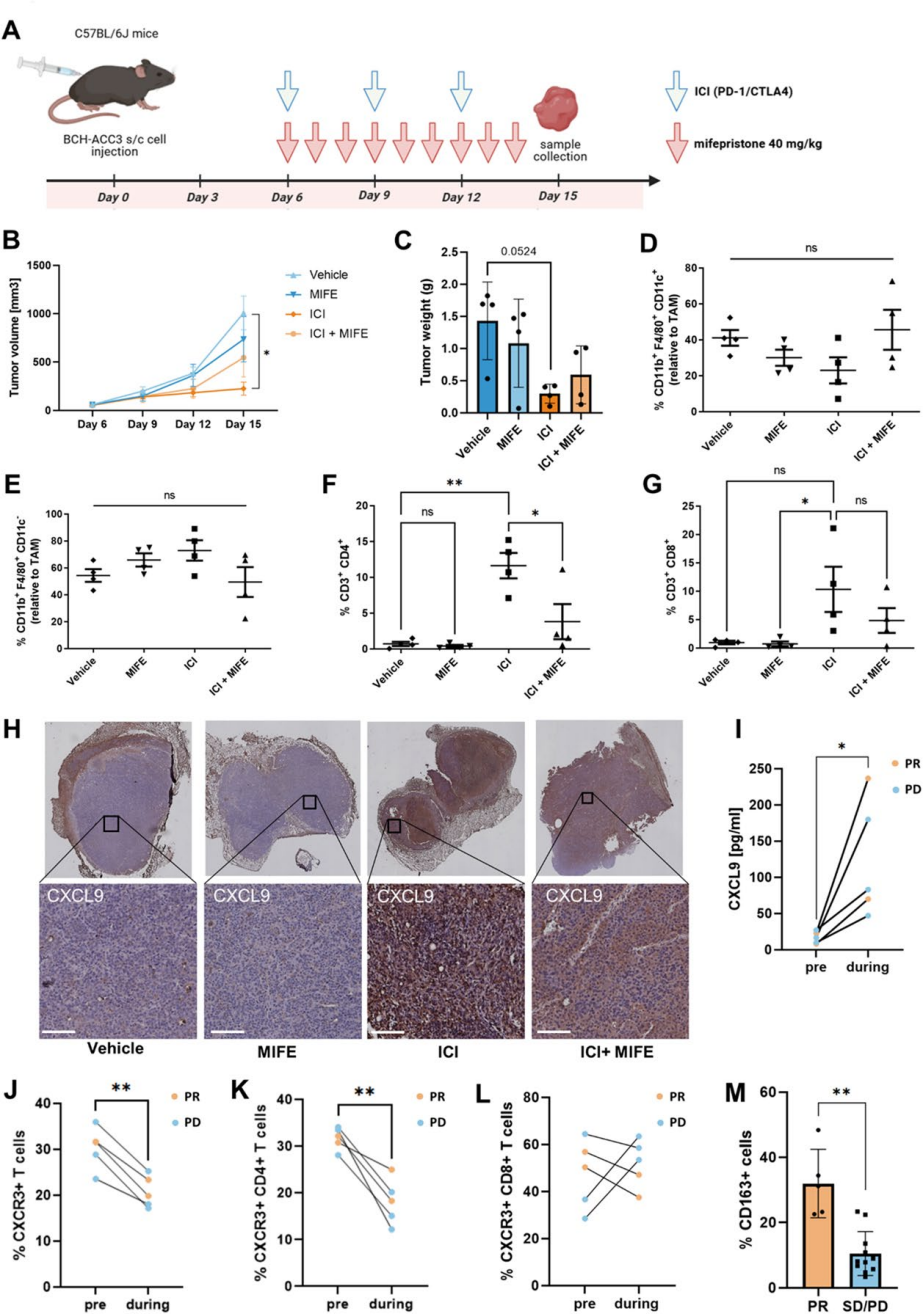
Rrole of TAMs in ICI efficacy in ACC. (**A**) Schedule for the subcutaneous (s.c.) BCH-ACC3 cell injection and treatment of established tumors with mifepristone (intraperitoneally (i.p.) at a dose of 40 mg/kg) and immune checkpoint inhibitor (ICI) cocktail of anti-PD1 antibody (200 ug per mouse) and anti-CTLA4 antibody (200 ug per mouse) **(B)** Tumor growth in the ACC model was evaluated by comparing tumor volumes on day 6, 12 and 15 post BCH-ACC3 cell injection. Each point of the graphic represents the mean ± SEM of four animals. Groups were compared using two-way ANOVA with Tukey’s multiple comparisons test. (**C**) Weight of ACC tumors on day 15 post BCH-ACC3 cell injection. Data is shown in mean +/- SD. Groups were compared using one-way ANOVA with Tukey’s multiple comparisons test. (**D**,** E**) Normalized percentage of tumor-infiltrating (D) CD11b+ F4/80 + CD11C+ cells (M1 macrophages) and (E) CD11b+ F4/80 + CD11C- cells (M2 macrophages) relative to total TAMs. Data is shown in mean +/- SEM. Groups were compared using one-way ANOVA with Tukey’s multiple comparisons test. (**F**,** G**) Percentage of tumor-infiltrating (F) CD4 + CD3+ T cells and (G) CD8 + CD3+ T cells. Data is shown in mean +/- SEM. Groups were compared using one-way ANOVA with Tukey’s multiple comparisons test. (**H**) Immunohistochemical staining of CXCL9 expression in paraffin-embedded tumors. Representative sections are enlarged. Scale bar: 100 µM. (**I**) CXCL9 concentrations in plasma samples of patients with ACC before and during ICI therapy. Differences between paired samples were assessed using paired t-test. (**J**) Percentages of CXCR3 + T cells in the peripheral blood of patients with ACC before and after the start of immunotherapy. Statistical comparisons were performed using paired t-test. (**K**) Percentages of CXCR3 + CD4+ T cells in the peripheral blood of patients with ACC before and after the start of immunotherapy. Statistical comparisons were performed using paired t-test. (**L**) Percentages of CXCR3 + CD8+ T cells in the peripheral blood of patients with ACC before and after the start of immunotherapy. Statistical comparisons were performed using paired t-test. (**M**) Percentages of CD163 + cells quantified by IHC staining of respective tissue samples of patients with ACC based on their response to ICI therapy. Differences were compared using Mann-Whitney-U-test. PR=partial response, PD=progressive disease; SD=stable disease.

In several cancers, the inhibition of steroid hormone synthesis or the GR has been proposed to enhance the response to immunotherapy by circumventing the immunosuppressive effects of glucocorticoids. Unexpectedly, our data showed a substantial reduction in the expression and secretion of the T cell chemoattractant CXCL9 upon GR blockade in macrophages using mifepristone, suggesting that targeting GR signaling in adrenal macrophages might impede ICI-driven T cell infiltration. Given these findings, we next investigated the efficacy of combined immune checkpoint inhibition (anti-PD-1 and anti-CTLA) and mifepristone in an ACC allograft model generated using the murine BCH-ACC3 cell line (Fig. 5A). This line was derived from a metastatic, glucocorticoid-producing tumor that arose in a BPCre mouse with adrenal-specific Wnt/β-catenin activation and p53 loss^24^. Treatment of ACC-bearing mice with the GR antagonist mifepristone alone showed a non-significant trend towards lower tumor growth, whereas ICI monotherapy markedly decreased ACC tumor growth (Fig. 5B, C). Intriguingly, combined treatment with ICI and mifepristone did not improve tumor growth inhibition but instead resulted in larger tumors compared to ICI monotherapy (Fig. 5B, C). To evaluate the impact of different immune cell subsets on the anti-tumor response, tumors were dissociated into single-cell suspensions and analyzed using multiparametric flow cytometry. Although not statistically significant, we observed a notable trend towards an increase in M1-like macrophage polarization (CD11b+ F4/80 + CD11c+) in the ICI+MIFE group compared to the ICI monotherapy group (Fig. 5D). In contrast, the percentage of M2-like macrophages (CD11b+ F4/80 + CD11c-) trended lower in the ICI+MIFE group compared to the ICI monotherapy (Fig. 5E). Interestingly, both tumoral CD4 + and CD8 + T cell infiltration was reduced in the combined treatment group, although this change was only statistically significant for CD4 + T cell infiltration (Fig. 5F, G). Analysis of paraffin-embedded tumors by immunohistochemical staining revealed strong CXCL9 expression in tumors isolated from ICI-treated mice, whereas CXCL9 staining was weaker in tumors of the combined treatment group (Fig. 5H). In summary, these experiments indicate that immunotherapy-driven tumoral CD4 + T cell infiltration may be hampered by concurrent treatment with mifepristone. This decreased CD4 + T cell infiltration may, in part, result from decreased CXCL9 production by mifepristone-exposed macrophages.

### In ACC patients, tumoral macrophage infiltration may serve as a biomarker for predicting immunotherapy response

Following these findings, we hypothesized that the presence of macrophages in ACC may be crucial for the attraction of CD4 + and CD8 + T cells to the tumor due to their potential to secrete large amounts of CXCL9. In line with this, in patients with ACC undergoing immune checkpoint inhibition therapy (clinical data in Table 1), plasma CXCL9 concentrations were significantly elevated after the initiation of immunotherapy (1–6 months) compared to baseline CXCL9 concentrations (0–3 months before ICI initiation) (Fig. 5I). We next analyzed PBMCs of these patients at the same time points. In both responders and non-responders, we observed a strong decrease in the percentage of T cells expressing CXCR3, the receptor for CXCL9, after the initiation of ICI therapy in comparison to baseline (Fig. 5J, Fig. S5A, B). This effect was primarily driven by a decrease in CXCR3 + CD4+ T cells (Fig. 5K). Interestingly, only in responders the percentage of peripheral CXCR3 + CD8+ T cells was substantially decreased during ICI treatment, while in non-responders this decline was less pronounced or percentages were even increased (Fig. 5L). Since we have demonstrated that macrophages polarized by glucocorticoids can produce excessive amounts of CXCL9 and given that macrophages are generally considered a primary source of this chemokine, we next assessed whether the proportion of TAMs (CD163 + cells) within the corresponding tumor tissue samples correlated with response to ICI therapy. In a larger cohort of ICI-treated patients with available tumor tissue sections, we observed that a higher percentage of intra-tumoral CD163 + cells at the time of resection was significantly associated with a response to ICI therapy (Fig. 5M). Together, these data suggest that the presence of C1Q+ CD163 + macrophages in GC-producing tissues, such as ACC, may improve immunotherapy outcomes through enhancing CXCL9-dependent CXCR3 + T cell chemoattraction in response to immunotherapy-induced IFNγ release.

**Table 1:** Clinical data of ICI treated patient; CR=complete response; PR=partial response; SD=stable disease; PD=progressive disease; GC=glucocorticoid; A=androgen.

Patients (*n*)	5
**Age at diagnosis**	48 (20–56)
**Sex** (n)	
Female	5
Male	0
**ENSAT stage at diagnosis** (n)	
I	0
II	1
of which responders to ICI	0
III	3
of which responders to ICI	2
IV	1
of which responders to ICI	0
**Compound** (n)	
Pembrolizumab	3
of which responders to ICI	2
Nivolumab	2
of which responders to ICI	0
**Best response to ICI** (n)	
CR	0
PR	2
SD	0
PD	3
**Clinical hormone excess** (n)	
inactive	0
GC	1
of which responders to ICI	0
A	1
of which responders to ICI	0
GC + A	3
of which responders to ICI	2
**Patients treated with mitotane before ICI** (n)	5
Mitotane parallel to ICI	1
of which responders to ICI	0

## Discussion

With the success of immunotherapies in many cancer entities, mechanisms that confer resistance and prevent treatment success have come into focus in molecular oncology. The ability of GCs to suppress T cell activity has been proposed to profoundly impact immunotherapy outcomes, but the actual consequences of GC action in the tumoral immune microenvironment are poorly understood^30,31^. To better characterize these local effects of glucocorticoids, we exploited the cell-autonomous hypersecretion of GCs present in the majority of ACCs. We show that tumor-associated macrophages dominate the tumor immune microenvironment of ACC irrespective of the extent of tumoral steroid hormone secretion. We find that GCs lead to a specific macrophage phenotype characterized by high expression of *CD163*, *MARCO*, and the complement component C1Q and a profoundly increased phagocytic capacity. Lastly, C1Q+ macrophages efficiently produce the chemokine CXCL9 upon IFNγ stimulation and may therefore be essential contributors to the success of ICI therapy.

In many cancer entities, tumoral T cell infiltration is considered one of the most crucial factors determining prognosis in cancer patients and the efficacy of immunotherapy^13,32–34^. In ACC, T cell infiltration has been reported to be limited, with a median of only 7.7 cells/HPF for CD3 + T cells^35^. Although an association between clinical cortisol excess and tumoral infiltration by CD4 + T cells has previously been shown, this association was not observed for CD8 + T cells^10,35^. In sharp contrast, an analysis of TCGA RNA data by *Baechle et al.*. revealed a significant association between clinically determined cortisol excess and CD8 + T cells, while no such association was found between cortisol excess and CD4 + T cells^36^. Hence, the role of tumoral GC secretion on the infiltration of ACC with different T cell subsets remains incompletely understood. Regarding ICI outcomes, GC excess at the time of diagnosis has not been significantly associated with differences in PFS or OS following ICI therapy^37^.Interestingly, a recent study reported an unexpected anti-tumor effect of low-dose dexamethasone in multiple cancer cell lines when co-cultured with T cells, as well as increased ICI sensitivity in a co-culture system, a 3D organoid model, and a humanized mouse model^38^.

TAMs play a critical role in shaping anti-tumoral immune responses^39^. In most cancers, macrophage infiltration increases with tumor stage and is associated with poor outcome, supporting a tumor-promoting role of TAMs^40^. In our ACC cohort, we observed a generally high abundance of intratumoral CD68 + and CD163 + cells in comparison to other tumor entities^41^. Notably, higher macrophage infiltration, as indicated by a high expression of CSF1 or C1QA, correlated with improved OS and PFS. Using both clinical data and tissue mass spectrometry of fresh-frozen ACC tissue specimens, we found no significant association between intratumoral macrophage infiltration and cortisol levels or established prognostic markers, suggesting that macrophages may exert an independent, potentially beneficial effect on patient outcome rather than merely reflecting associations with factors such as cortisol secretion or tumor stage. In contrast to our results, a deconvolution analysis of TCGA data by Baechle *et al.* observed a small, yet significantly increased presence of M1 macrophages and a non-significant trend towards more M2 macrophages in non-cortisol-secreting ACCs^36^. Meanwhile, Wilmouth *et al.* found that infiltration by phagocytic macrophages (CD68/TREM2/TYROBP gene signature) was more frequent in men than in women and was associated with a better prognosis, but not with patients’ hormonal status^42^.

Our RNA expression analysis of *in vitro* polarized MΦ revealed a robust glucocorticoid-driven CD163 + C1Q+ macrophage phenotype, induced either by dexamethasone or by CM from the steroidogenic ACC cell line NCI-H295R. Compared with dexamethasone-conditioned macrophages, the effects induced by ACC cell-CM were less pronounced (Fig. S6A, B), which is consistent with the cortisol concentrations measured in the conditioned medium (10 µg/dL; Fig. S2E), corresponding to ~ 276 nM cortisol and an estimated GC activity equivalent to approximately 10 nM dexamethasone (assuming an 25-fold higher potency of dexamethasone compared to cortisol). The presence of a C1Q+ macrophage phenotype has previously been identified in other solid cancers, including melanoma, breast cancer, and hepatocellular carcinoma^43–45^. In these tumor entities, we found a significantly increased *C1QA/CD68* gene expression ratio in tumor samples with high *HSD11B1* expression, supporting a link between local GC production and C1Q+ macrophages in other tumor microenvironments. Still, current knowledge on the impact of C1Q+ macrophages on T cell function and patient prognosis is ambiguous, highlighting their complex and context-specific roles. A recent multi-omics study identified a PD-L1+/MHCII+/C1Q+ macrophage phenotype that is immunostimulatory to T cells and associated with favorable clinical outcomes in breast cancer patients^46^. Additionally, a FOLR2 + TAM population (identified by high levels of *APOE*, *APOC1*, *C1QA*, and *C1QC*) was identified to be associated with CD8 + T cell infiltration in the same tumor entity^47^. On the other hand, the presence of tumoral (secreted) C1q has also been linked to tumor growth promotion in two distinct mouse models^44,48^, pinpointing towards a pro-tumoral role of C1Q+ macrophages in some tumor entities. In our study, we did not observe that C1q treatment of ACC cells affected their proliferation over 48 h (Fig. S7A). Moreover, the proliferation of CD8 + T cells in the presence or absence of CD3/CD28 activation remained unaltered when exposed to either C1Q+ MΦ or M1-polarized macrophage supernatant (Fig. S7B).

We here demonstrate that GC-induced C1Q+ macrophages are capable of producing high amounts of the T cell chemoattractant CXCL9 in response to IFNγ, a hallmark of ICI therapy^49^. This ligand of the CXCR3 chemokine system has already been shown to be required for the efficacy of anti-PD-1 therapy in several cancer models^50–53^. Most commonly, CXCL9 secretion is associated with a pro-inflammatory M1-like macrophage phenotype^52,54^. For example, in an *in vivo* model of breast cancer, macrophages (CD11b+ Ly6Cint CD11c+ F4/80+) were defined as the predominant producers of CXCL9 and were essential for dual PD-1/CTLA4 therapeutic efficacy^52^. However, recent work suggests that TAM polarization is better reflected by a CXCL9/SPP1 axis than by conventional M1/M2 markers. In HNSCC, a high CXCL9:SPP1 ratio (CXCL9^hi^ TAMs) correlated with improved clinical outcome and response to anti–PD-1–based therapy, and the authors propose that multiple TAM sublineages can engage this axis to varying degrees in response to extracellular cues^55^. Interestingly, our dexamethasone-conditioned macrophages also showed increased CXCL9 expression and reduced SPP1 expression after IFNγ stimulation compared to M1-polarized controls (Fig. S6A). Moreover, ACC-bearing mice treated with dual PD-1/CTLA4 blockade displayed increased tumoral CXCL9 expression and enhanced CD4⁺ and CD8⁺ T cell infiltration. This effect was attenuated by concurrent mifepristone treatment, despite an increased abundance of CD11b⁺ CD11c⁺ F4/80⁺ “M1” macrophages, further supporting a potential role for GC-polarized macrophages in shaping the response to ICI therapy. Nonetheless, since mifepristone also acts as an antagonist of the progesterone receptor—and given that progesterone can also potently influence tumoral immune responses [56]  —we cannot exclude the possibility that the observed effects are, at least in part, mediated through the blockade of progesterone receptor signaling. Additionally, the gating strategy for murine macrophages did not include C1Q as a marker for GC-polarized macrophages. Therefore, validation of the contribution of the CXCL9/CXCR3 axis in an in vivo model of ACC warrants further investigation in future studies.

In a small cohort of five patients with ACC receiving ICI therapy, we observed that CXCL9 levels increased markedly after the start of anti-PD1 therapy. Moreover, a high proportion of CD163 + macrophages was significantly associated with response to ICI therapy. Similarly, a recently published study on the efficacy of the PD-1 inhibitor camrelizumab in combination with apatinib in pre-treated ACC patients revealed a connection between the response to this therapy regimen and the presence of baseline CXCR3 + CD8+ T cells^10^, underlining the relevance of macrophage-produced CXCL9 on the CXCR3-dependent T cell chemoattraction in ACC. Nonetheless, compared with their results, we did not find basal percentages of CXCR3 + CD8+ T cells to predict the response to ICI monotherapy; rather, we found that a decrease in peripheral CXCR3 + CD8+ T cells after ICI initiation may predict response. This suggests that, in particular, the migration of CXCR3 + CD8+ T cells to the tumor site may be a key contributor to the response to anti-PD1 therapy in these patients.

Our data suggest that C1q-mediated phagocytosis plays a central role in the tumoricidal activity of macrophages in ACC. Consistent with this, a highly phagocytic macrophage phenotype was recently reported in a spontaneous ACC model in male mice^42,57^. In this study, bulk RNA sequencing of tumors from ACC-bearing mice showed a marked up-regulation of *C1QA/B/C, TREM2 and MERTK expression*^42^. Notably, these genes were primarily expressed by macrophages based on sc-RNA-seq analysis of WT mouse adrenals, closely aligning with the macrophage phenotype observed in our study. However, in the context of cancer, phagocytosis of tumor cells may be a double-edged sword. On the one hand, the clearance of tumor cells and the presentation of tumor antigens can enhance the adaptive antitumor immune response. On the other hand, clearance of cancer cells may prevent the release of damage-associated molecular patterns, thus contributing to immune evasion and tumor progression. Undoubtedly, unraveling the consequences of macrophage-mediated tumor cell clearance in this context requires further investigation.

Undeniably, our study has certain limitations. One of the main restrictions is the small number of clinically annotated patient samples. This is primarily due to the rarity of this endocrine malignancy. In addition, analyses of patients with ACC undergoing immunotherapy were performed retrospectively; therefore, the availability of material from a large number of patients at the relevant timepoints was limited. Further, in the majority of cases, different treatments, particularly mitotane, took place between tumor resection and the initiation of ICI therapy, preventing us from accurately evaluating TAM infiltration directly preceding ICI administration. Moreover, the well-established negative impact of systemic GC excess on patient outcomes^35,58^ may seem contradictory to the favorable impact of GC-induced C1Q+ macrophages observed in this study. However, our data also underlines that C1Q+ macrophage polarization occurs under physiological cortisol concentrations within the adrenal gland and in ACCs with lower expression of steroidogenic enzymes, suggesting that the detrimental systemic impact of GC excess in some ACC patients may outweigh the apparent beneficial impact of C1Q+ macrophages on tumor control. Furthermore, a major limitation of the current study is its strong reliance on *in vitro*–generated macrophages. In addition, most of the mouse experiments did not reach statistical significance, likely due to the limited number of animals included.

Despite these open questions, the results obtained in this study contribute to a better understanding and characterization of the GC-induced C1Q+ macrophage phenotype in ACC and potentially other tumor entities, particularly in the context of immunotherapies. Our results highlight two important points. First, therapies that inhibit macrophages (such as CSF1R targeting therapies) may have to be used very cautiously in GC-producing tumor entities, especially when combined with T cell-based treatments (e.g., CAR-T or ICI). Depletion of C1Q+ macrophages may eradicate key mediators of tumor cell clearance, e.g., by efficient phagocytosis and crucial facilitators of T cell infiltration, a major determinant of immunotherapy efficacy. Second, assessing the quantity of macrophages within ACC tumor tissues could potentially serve as a useful indicator to predict the response to ICI therapy.

## Electronic Supplementary Material

Below is the link to the electronic supplementary material.

Supplementary Material 1

Supplementary Material 2

Supplementary Material 3

## Data Availability

The datasets generated and analyzed during the current study are available through NCBI’s Gene Expression Omnibus under GEO Series accession numbers GSE329681 (https://www.ncbi.nlm.nih.gov/geo/query/acc.cgi?acc=GSE329681), GSE329772 (https://www.ncbi.nlm.nih.gov/geo/query/acc.cgi?acc=GSE329772), and GSE329775 (https://www.ncbi.nlm.nih.gov/geo/query/acc.cgi?acc=GSE329775). The use of the THP-1 monocytic line expressing Cas9 is covered by a material transfer agreement (MTA) between Prof. Dr. Jörg Wischhusen and Dr. Isabel Weigand.

## Abbreviations

ACC: adrenocortical carcinoma
CXCL9: CXC motif chemokine ligand 9
ENSAT: European Network for the Study of Adrenal Tumours
FDR: false discovery rate
GC(s): glucocorticoid(s)
GM-CSF: Granulocyte macrophage-colony stimulating factor
GR: glucocorticoid receptor
HC: hydrocortisone
HPF: High Power Field
ICI: immune checkpoint inhibition
IF: immunofluorescence
IHC: immunohistochemistry
Lip-1: Liproxstatin 1
MΦ: macrophage
M-CSF: macrophage-colony stimulating factor
OS: overall survival
PBMC: peripheral blood mononuclear cell
PFA: paraformaldehyde
PFS: progression-free survival
PMA: phorbol-12-myristate-13-acetate
PS: phosphatidylserine
RSL3: (1 S, 3R) Ras-selective lethal small molecule 3
TAM: tumor-associated macrophage
TME: tumor microenvironment
WB: western blot
