# Supplementary material for: Glucocorticoids induce a phagocytic C1Q+ macrophage phenotype primed for IFNγ-dependent CXCL9 secretion
Source: Sci Rep. 2026 May 18;16:15345. doi: 10.1038/s41598-026-52733-y (PMC13183869; doi:10.1038/s41598-026-52733-y)

Figure 1F: CD206

Ladder: PageRulerTM Plus Prestained Protein Ladder; Product#26619

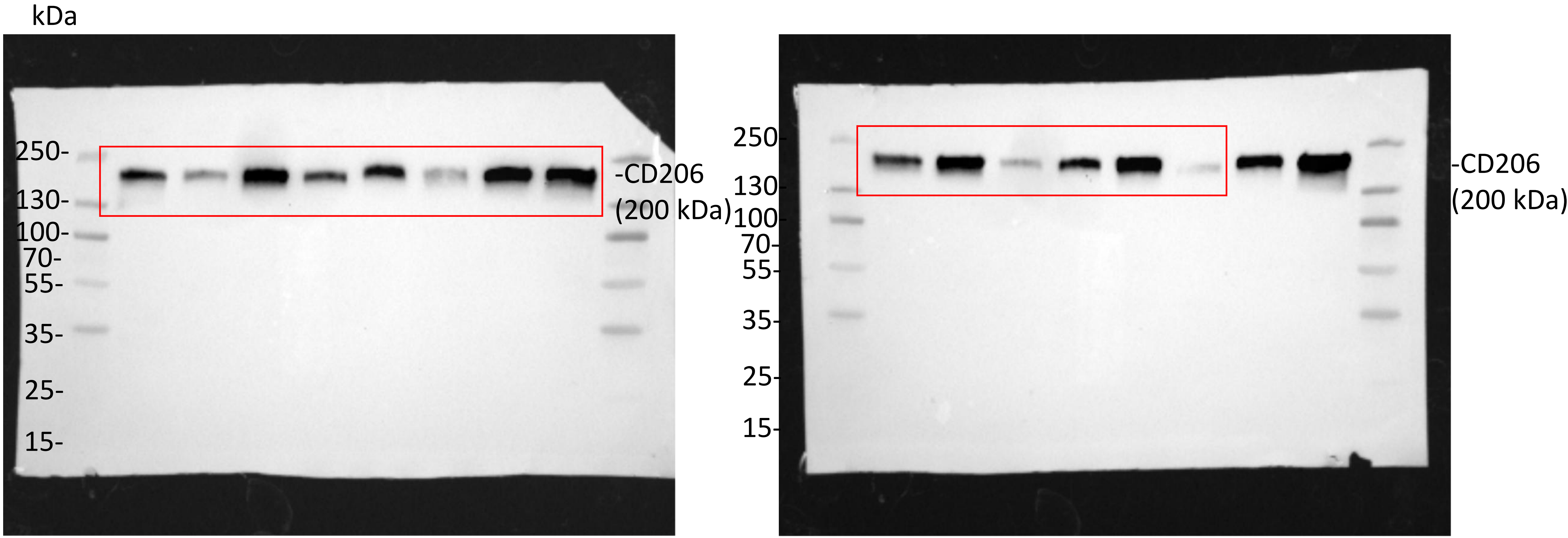

Figure 1F: CD163

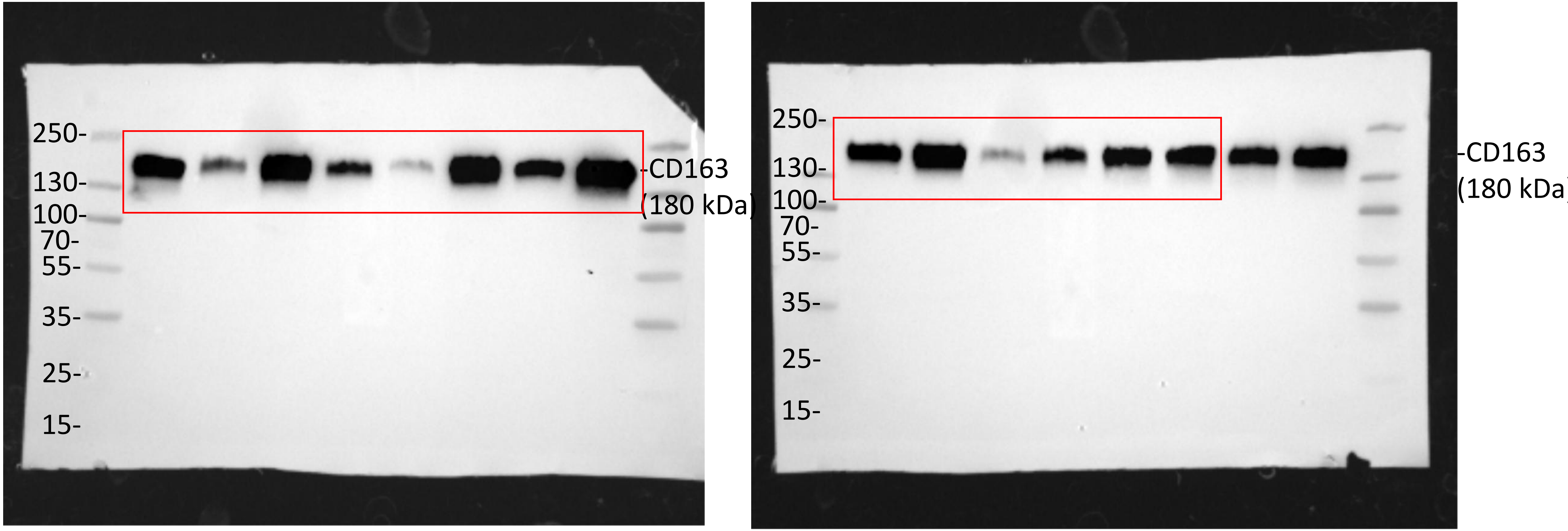

Figure 1F: GAPDH

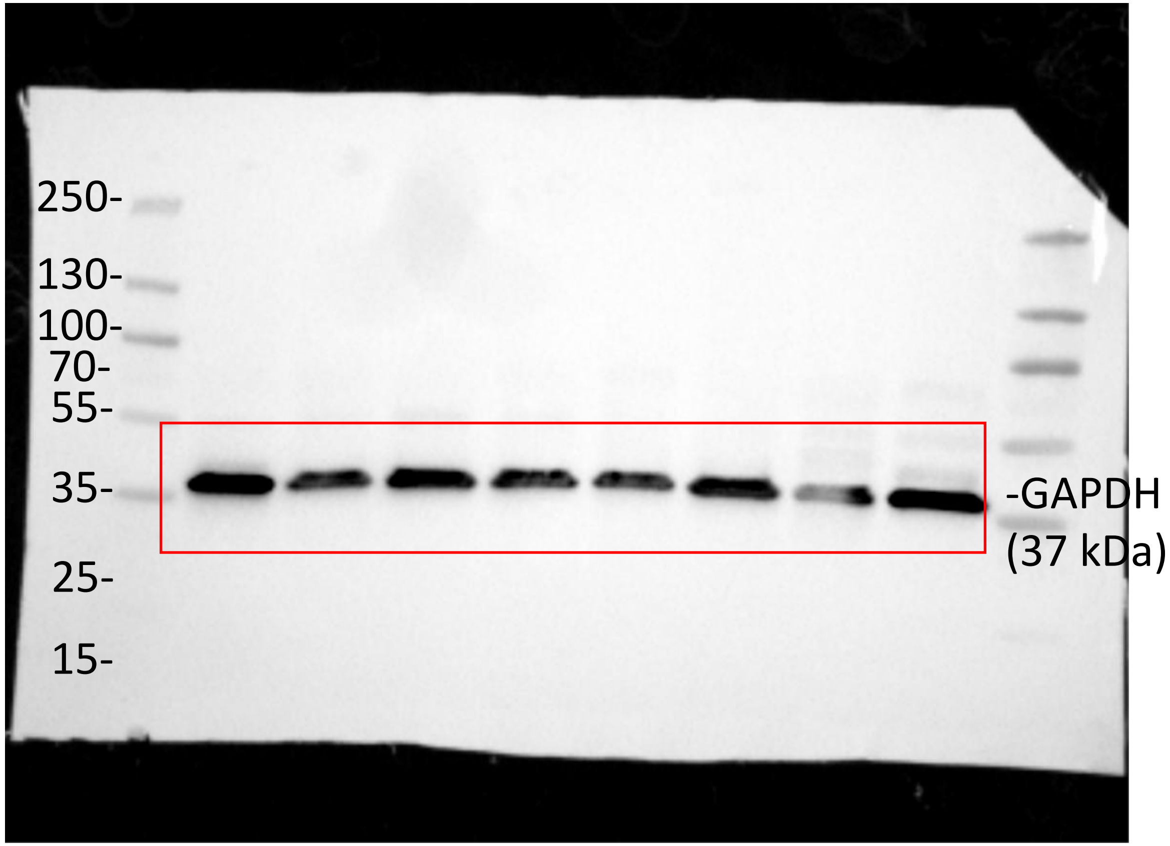

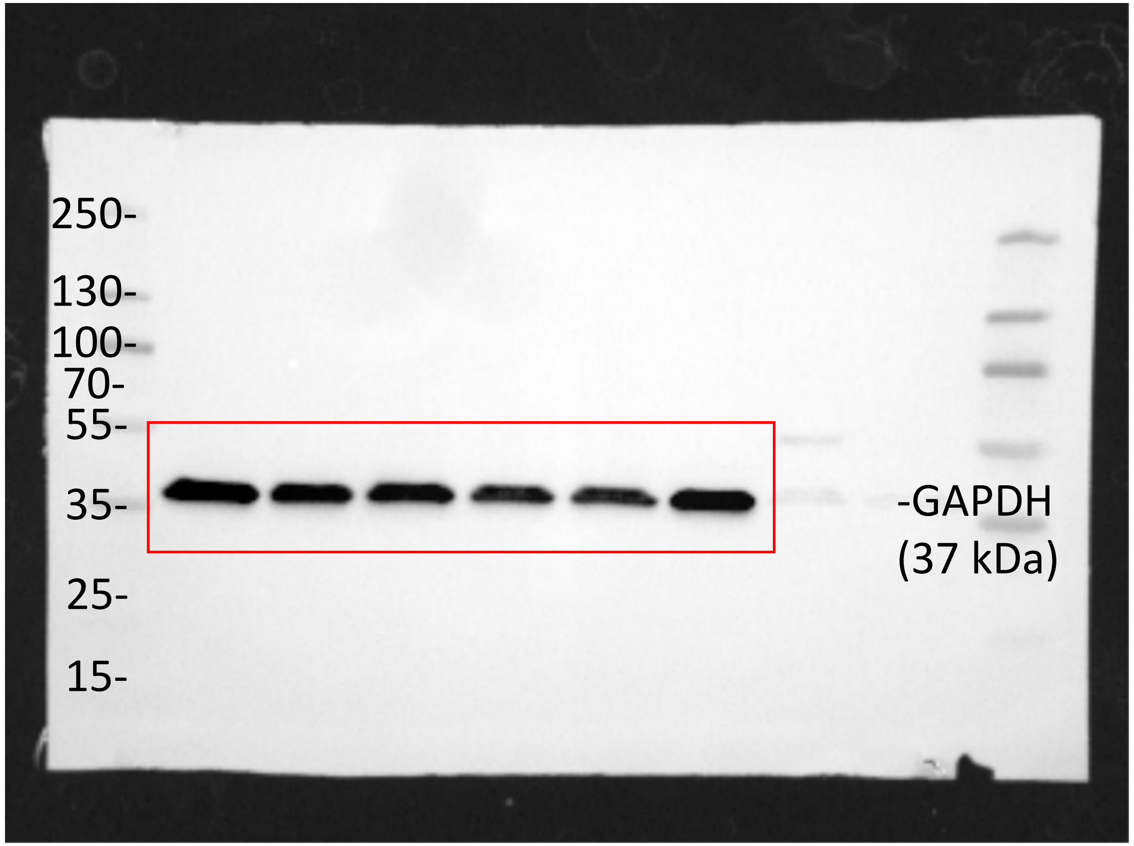

Figure 2I:

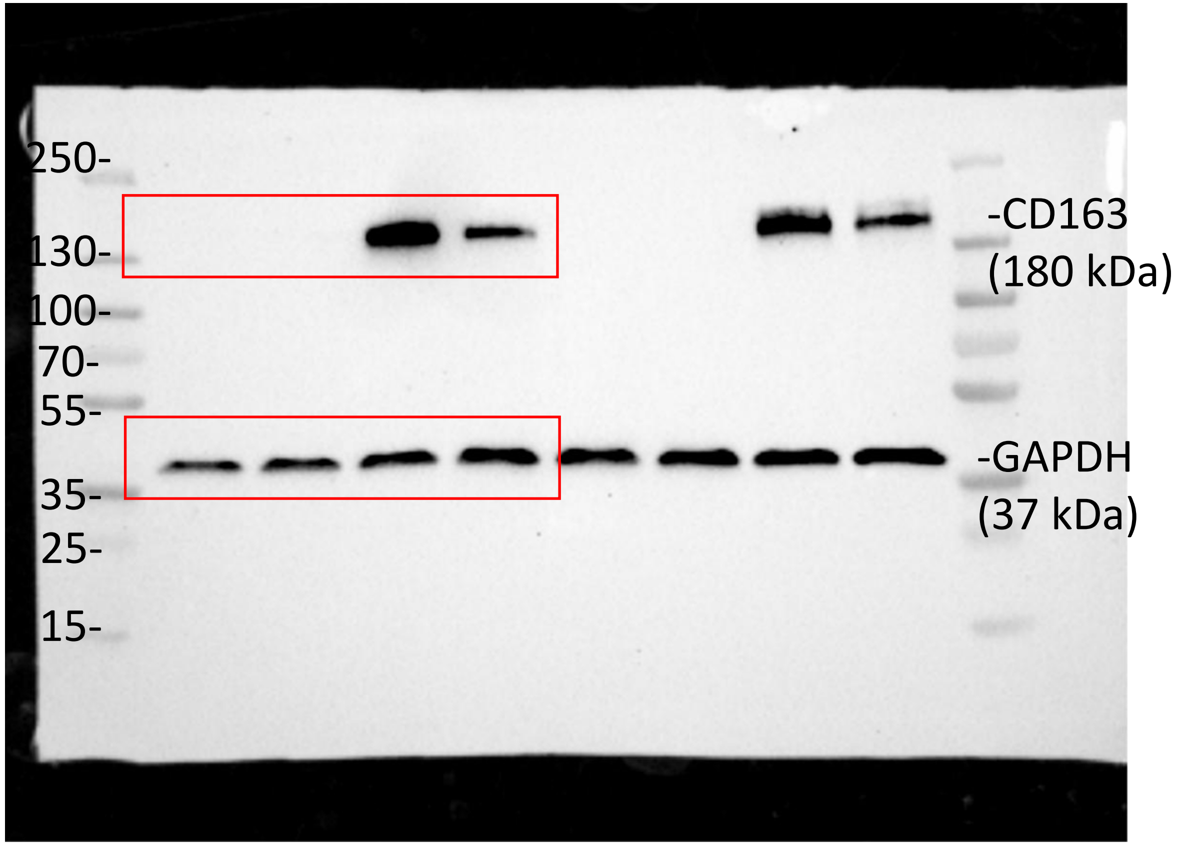

Figure 2I:

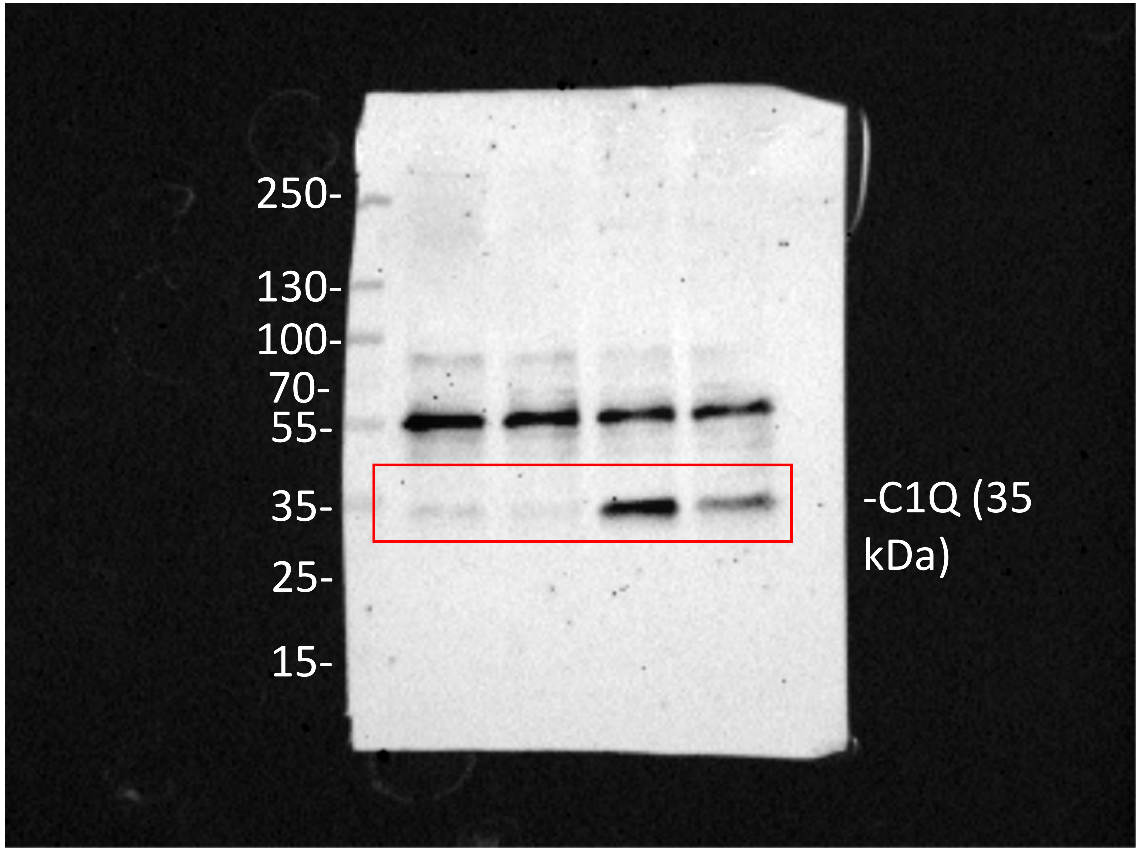

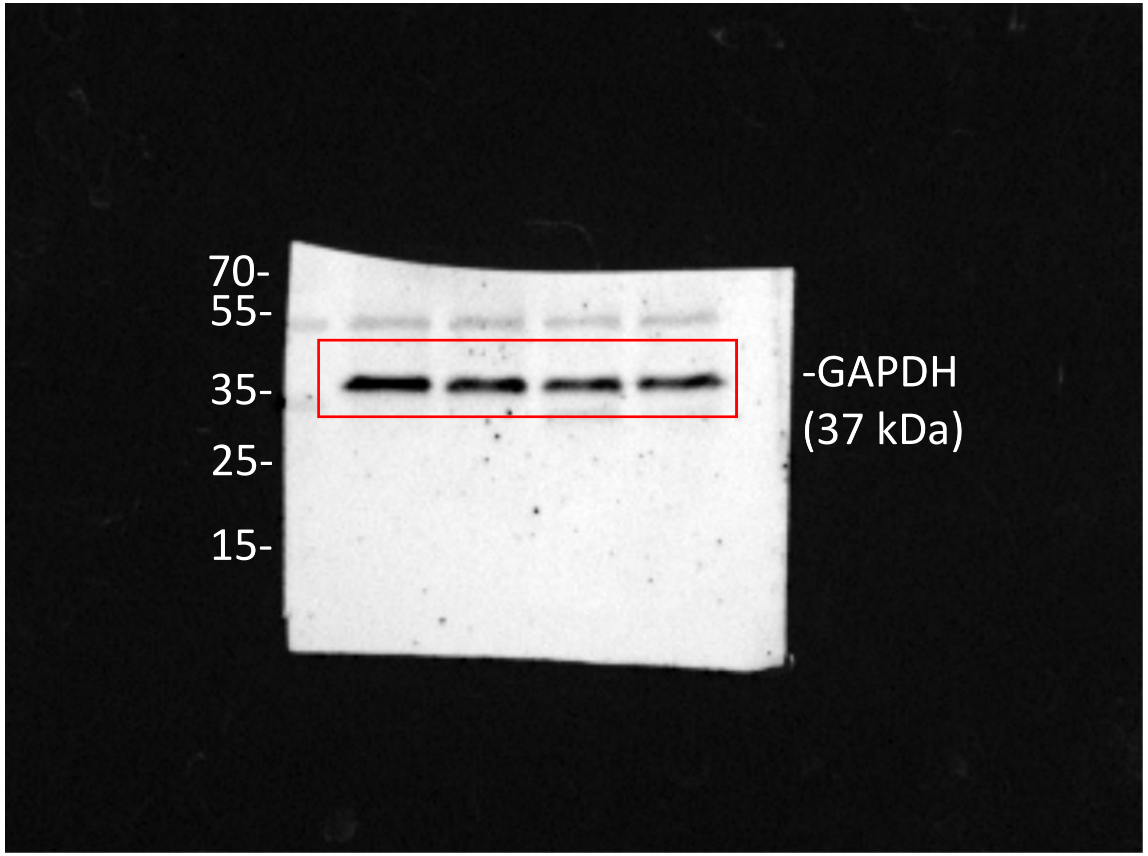

Figure 2J:

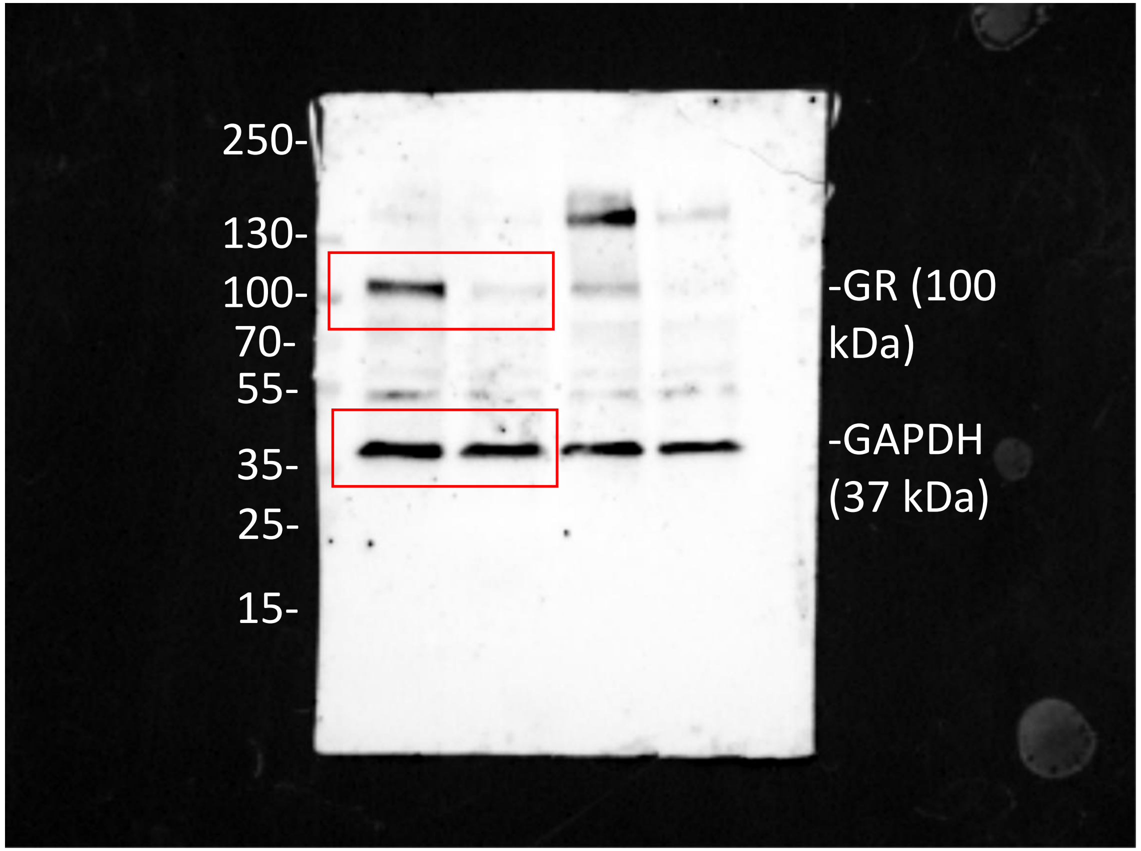

Figure 2K:

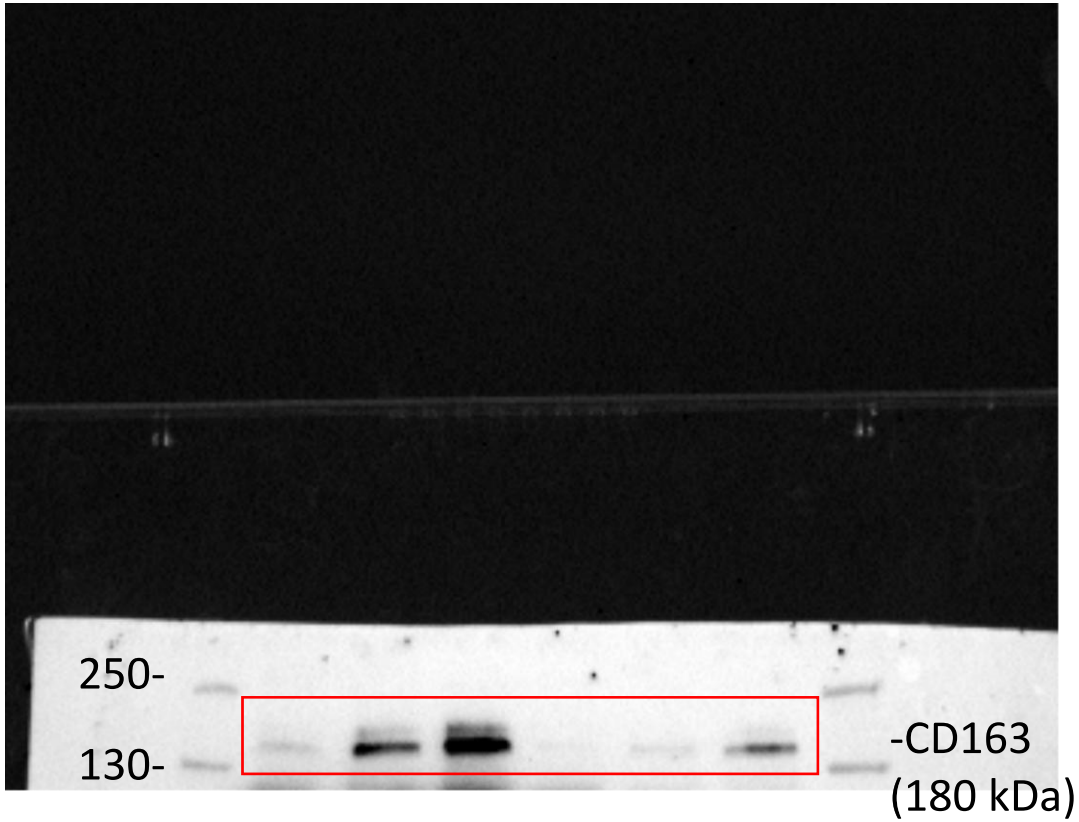

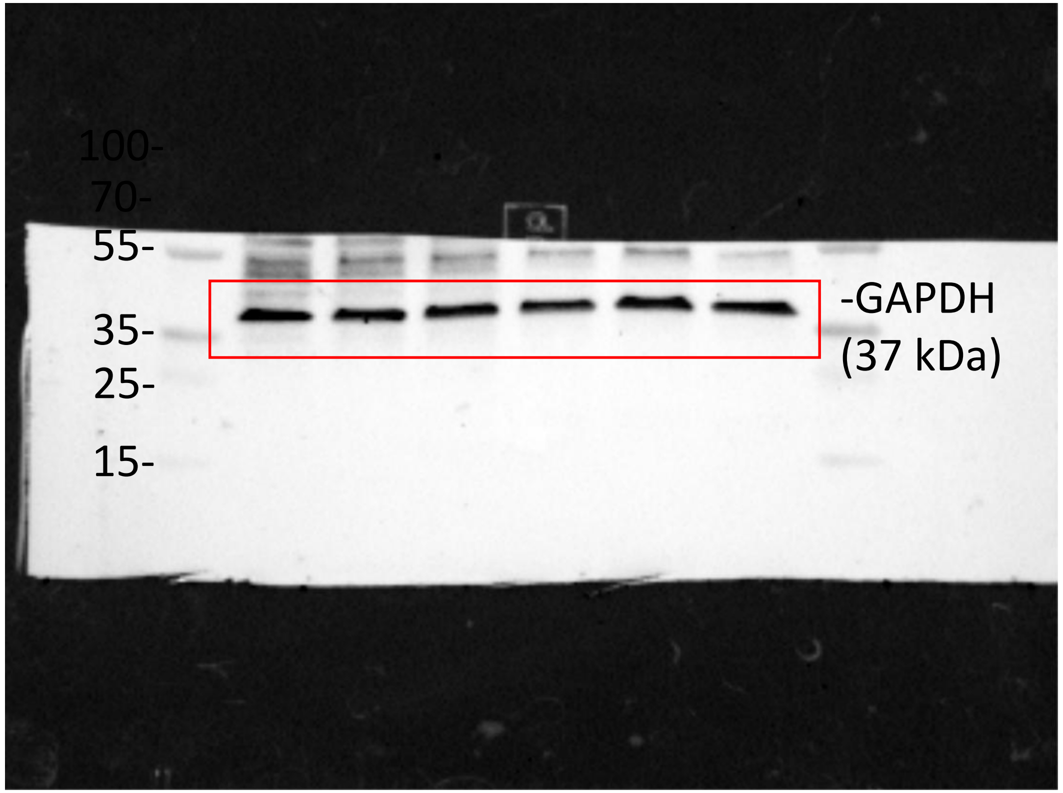

Figure 4E:

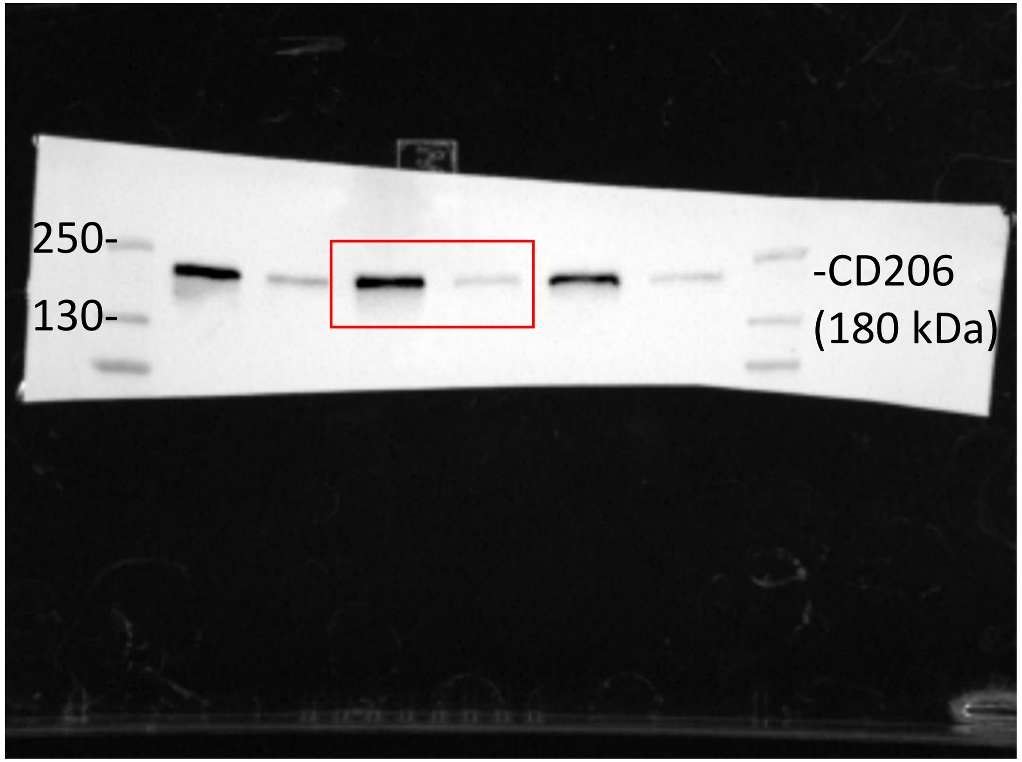

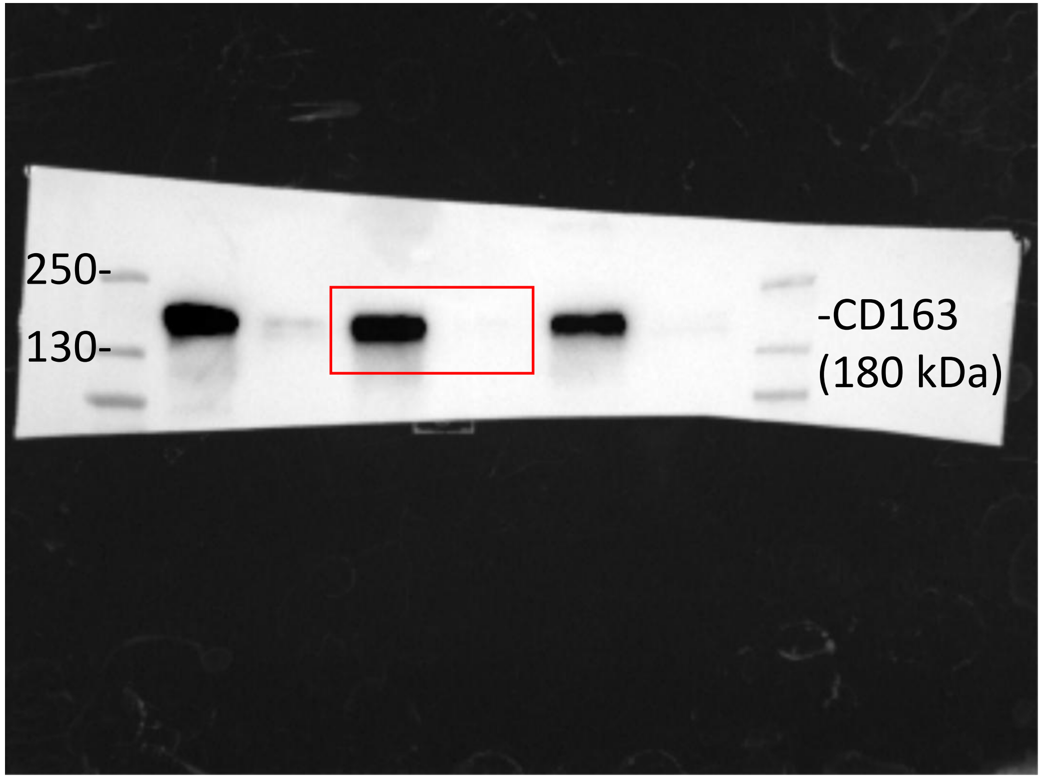

Figure 4E:

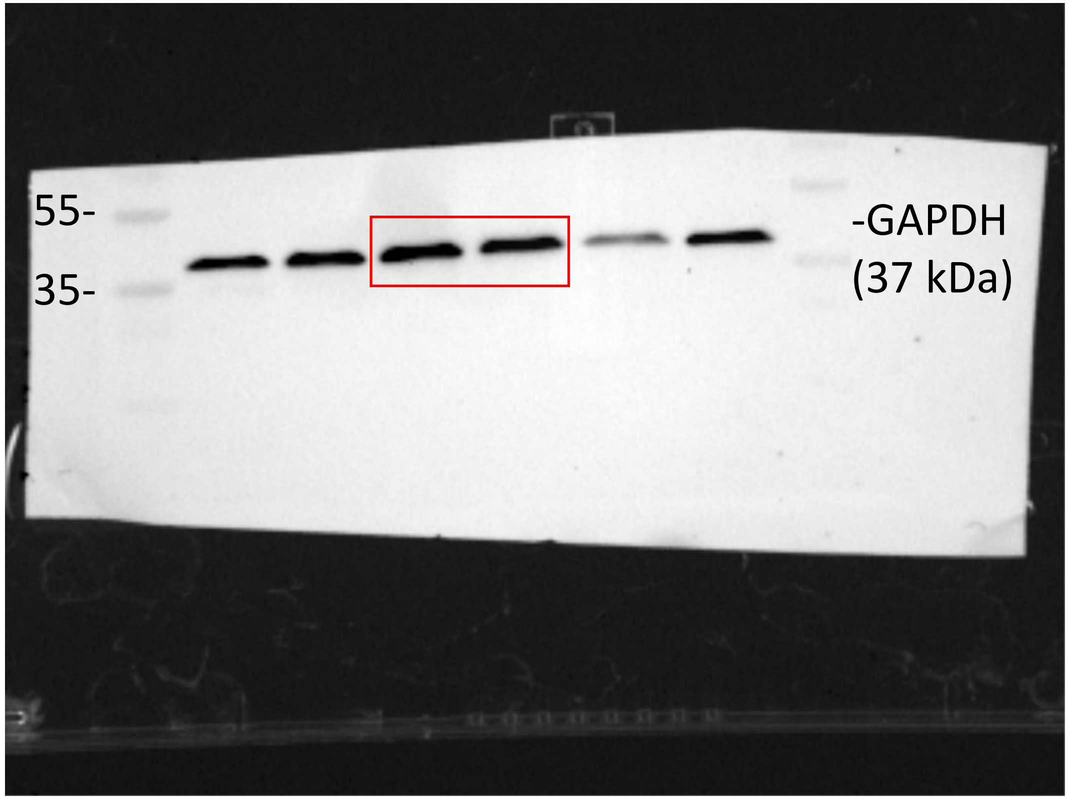

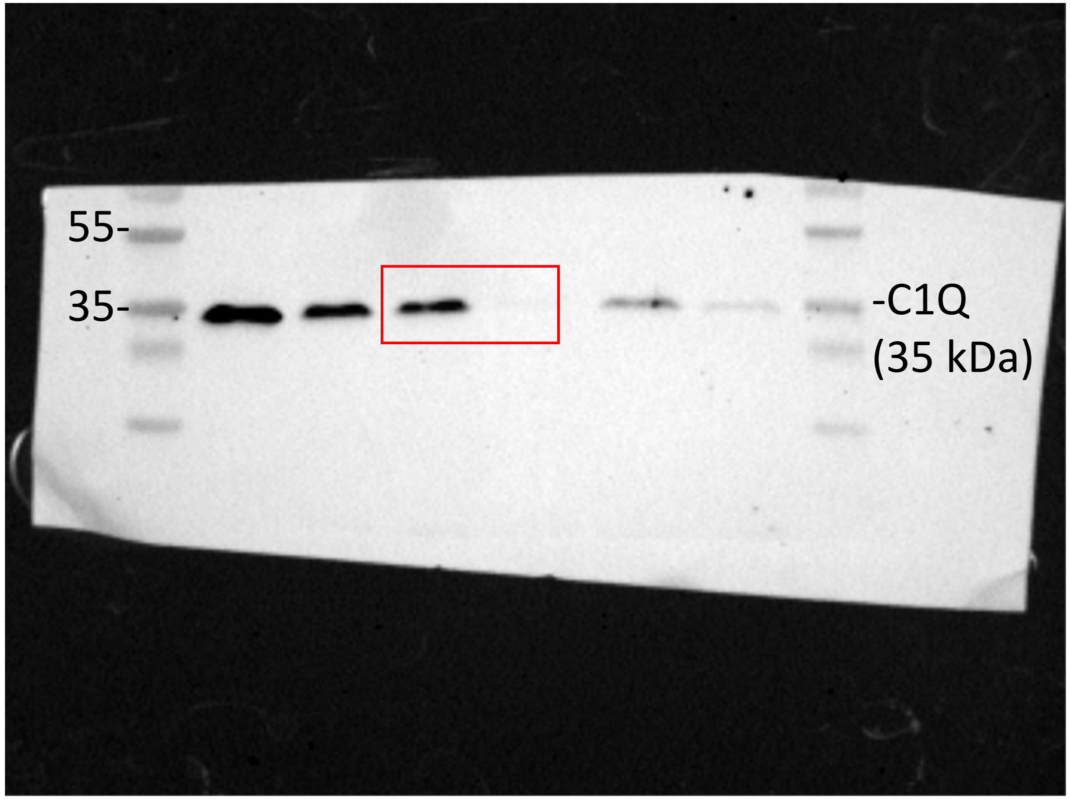

Figure S2A:

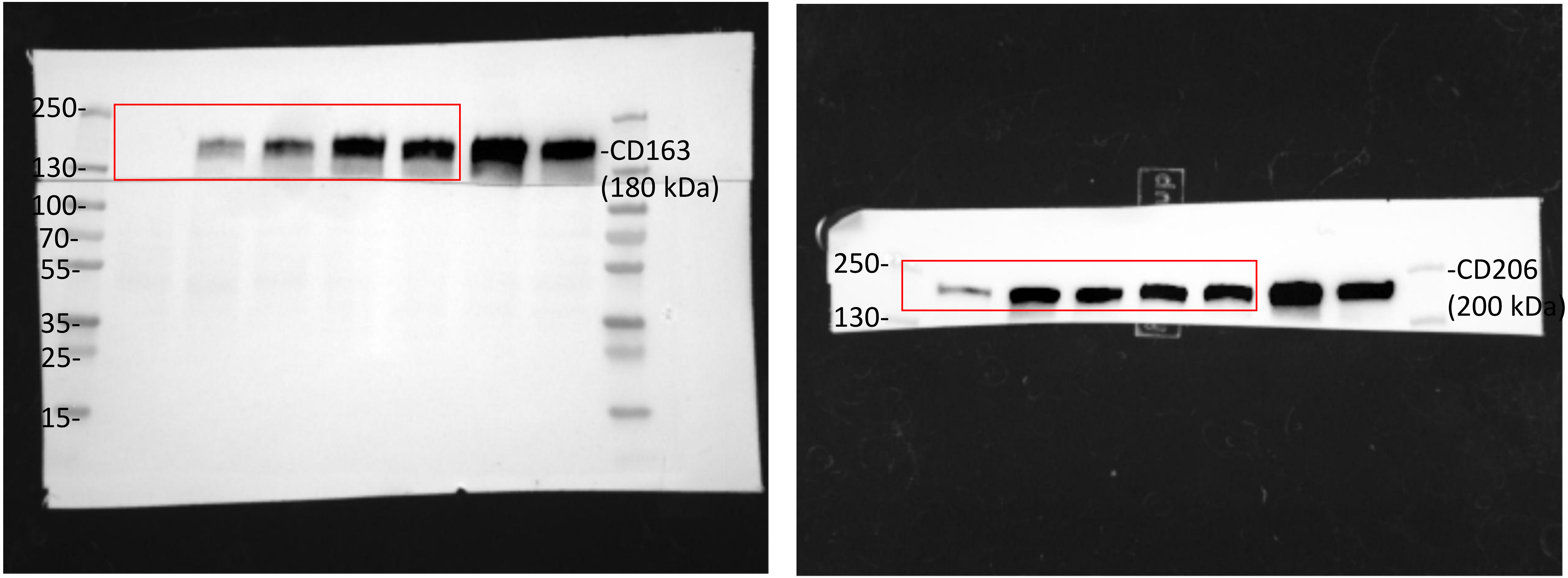

Figure S2A:

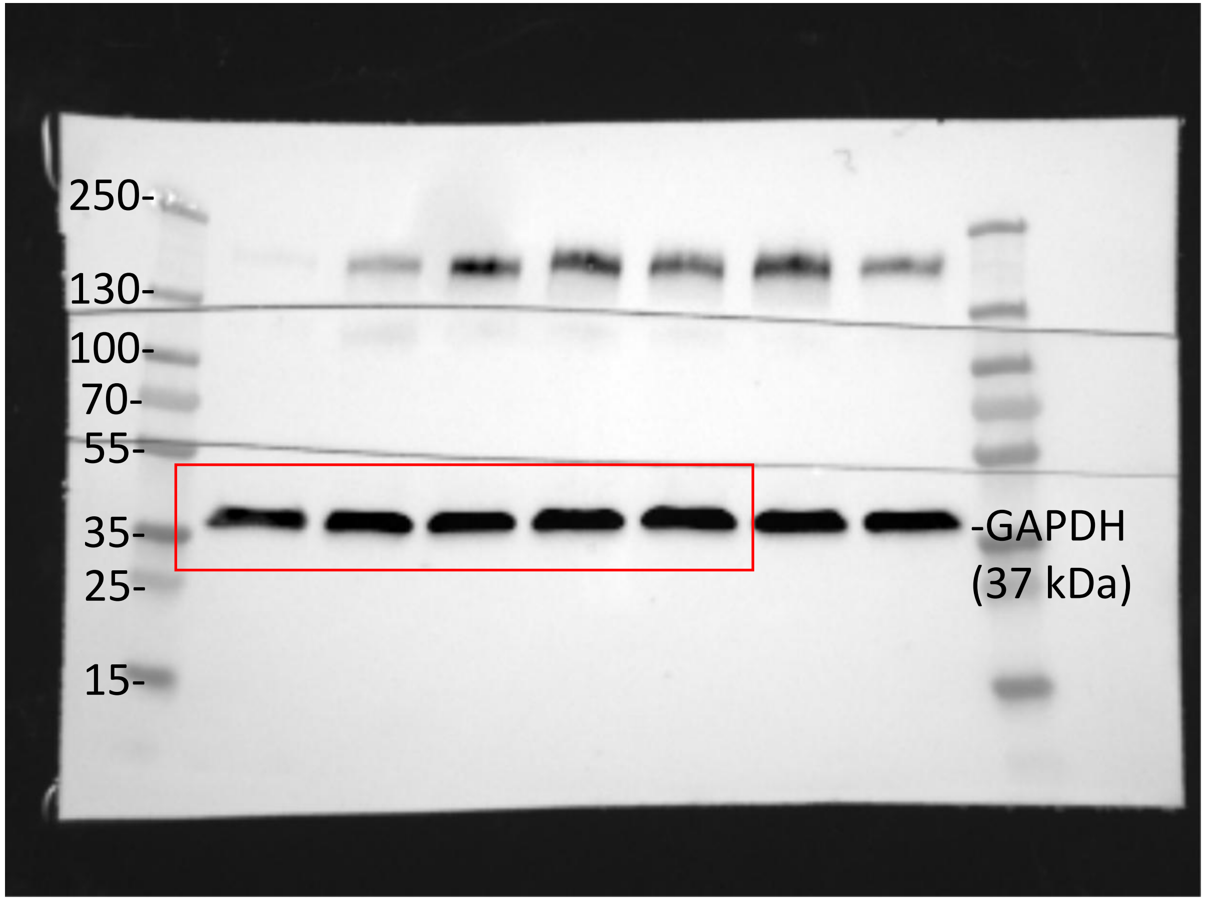

Figure S3F:

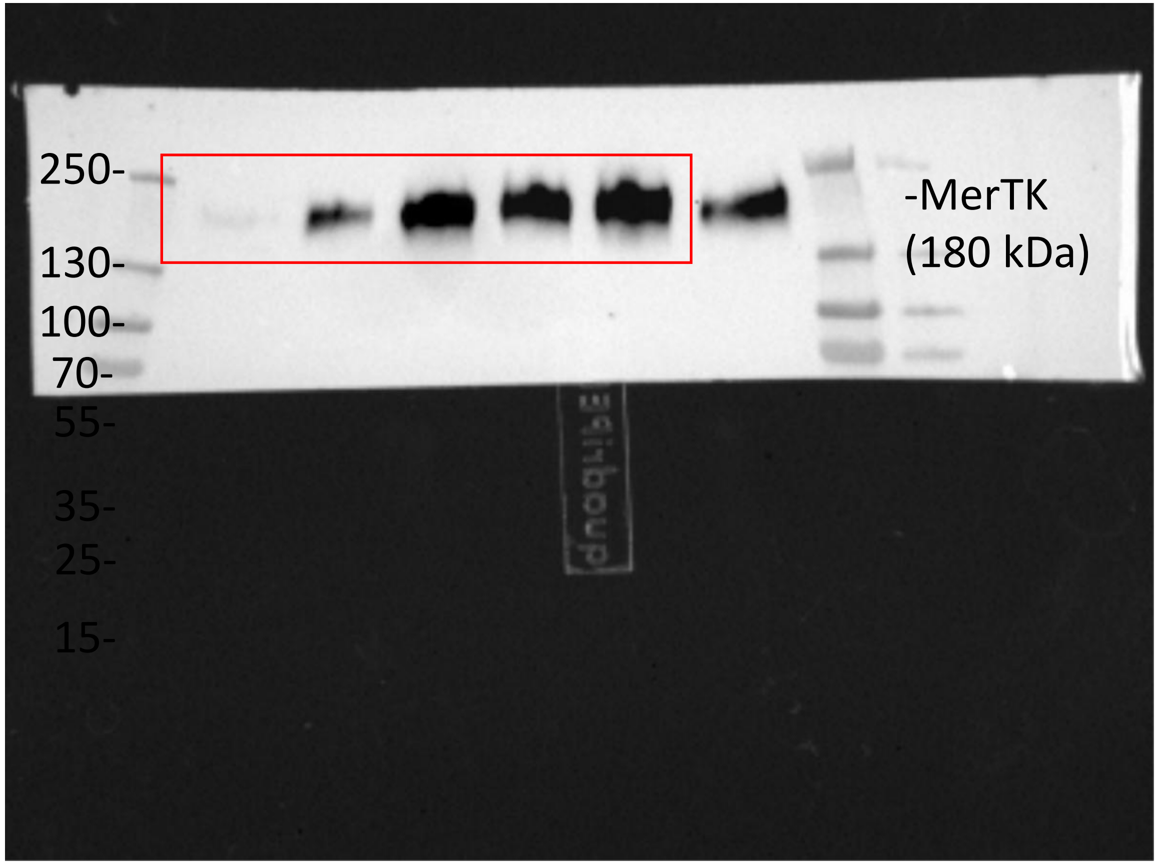
250-
130-
100-
70-
55-
35-
25-
15-
-MerTK
(180 kDa)

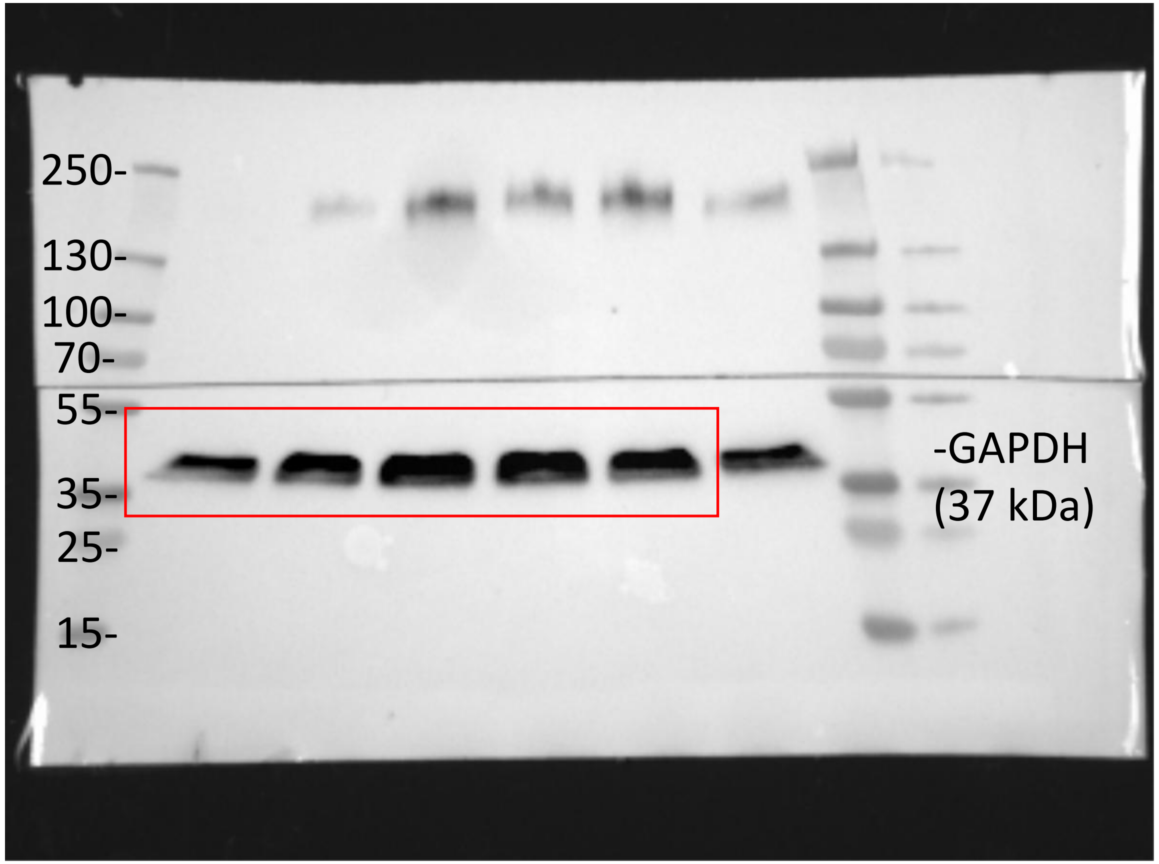
250-
130-
100-
70-
55-
35-
25-
15-
-GAPDH
(37 kDa)

Figure S3F:

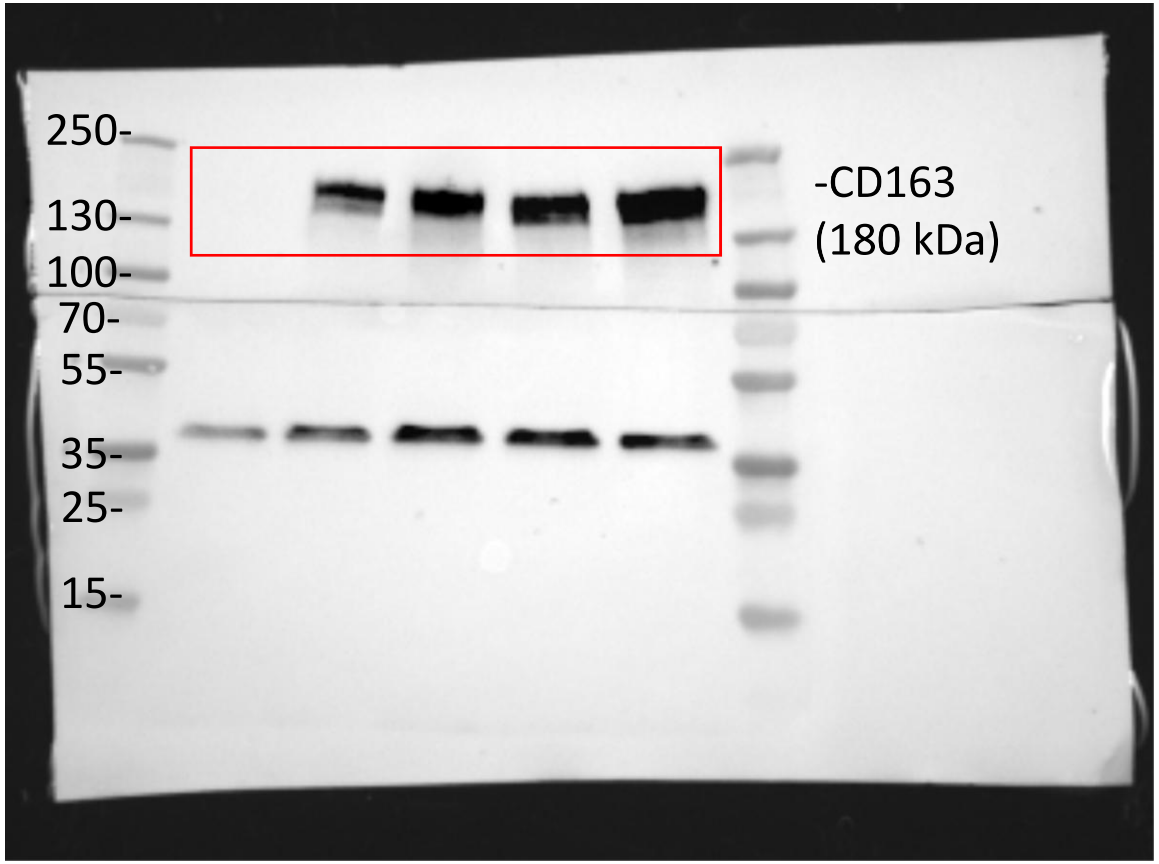
250-
130-
100-
70-
55-
35-
25-
15-
-CD163
(180 kDa)

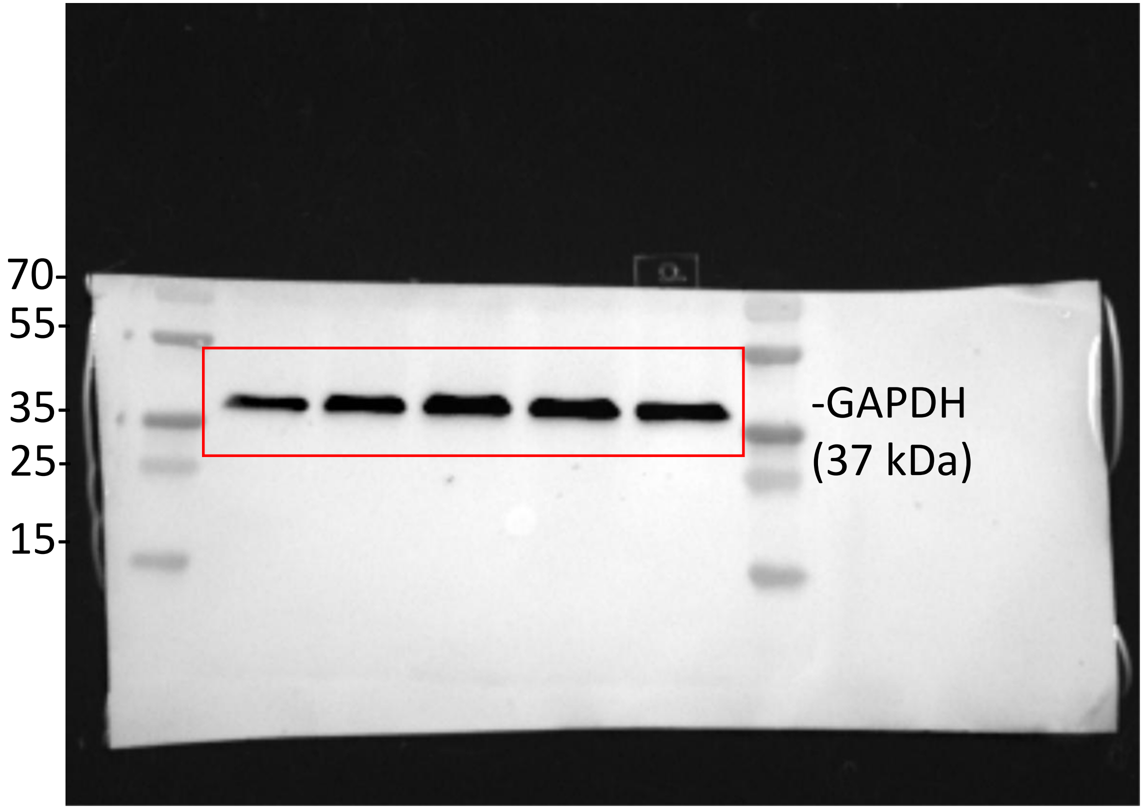
70-
55-
35-
25-
15-
-GAPDH
(37 kDa)

Figure S4A:

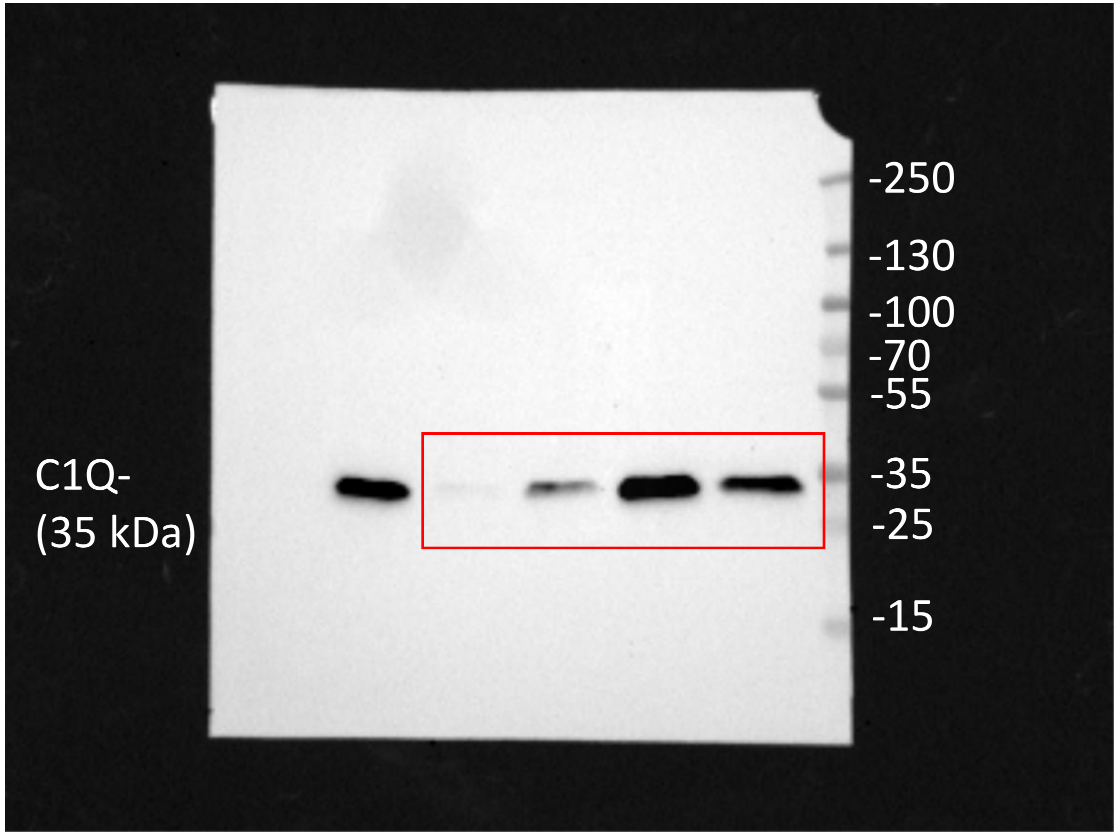

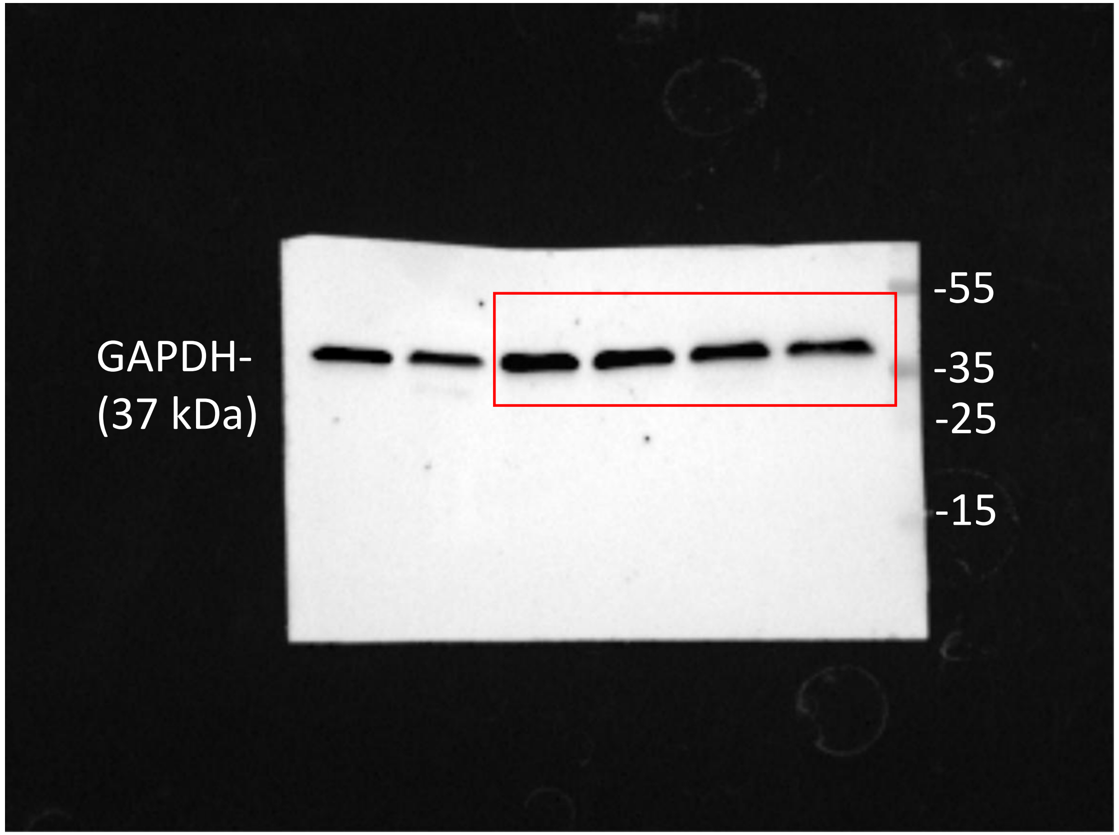

Supplement: Supplementary file 1 — Supplementary Material 1 [file 41598_2026_52733_MOESM1_ESM.pdf]
