# Supplementary material for: Glucocorticoids induce a phagocytic C1Q+ macrophage phenotype primed for IFNγ-dependent CXCL9 secretion
Source: Sci Rep. 2026 May 18;16:15345. doi: 10.1038/s41598-026-52733-y (PMC13183869; doi:10.1038/s41598-026-52733-y)
Supplement: Supplementary file 3 — Supplementary Material 3 [file 41598_2026_52733_MOESM3_ESM.pdf]

Supplementary methods

T cell isolation

Human PBMCs from healthy blood donors were isolated by density gradient using Lymphoprep™ (Stemcell). CD8+ T cells were isolated using the EasySep™ CD8+ T Cell isolation kit (#17953) and cultured in ImmunoCult™-XF T Cell Expansion medium supplemented with 20 ng/ml IL-2 and 25 µl/ml ImmunoCult™ Human CD3/CD28 activator (all Stemcell)

Seahorse metabolic phenotyping

At the day of the assay, MΦ were washed with PBS and Seahorse XF RPMI medium, pH 7.4 (Agilent, 103576) supplemented with 2 mM L-Glutamine. The metabolic flux was measured using the Agilent Seahorse XFe96 Analyzer (RRID:SCR_019545) according to the manufacturer's protocol. For the metabolic rate test, the following reagents were injected in sequential order for resulting final concentrations: A) 5 mM Glucose; B) 1.5 µM Oligomycin (Sigma Aldrich, 495455); C) 2.5 µM Bam15 (Sigma Aldrich, SML1760-5MG); D) 50 mM 2-DG (Sigma Aldrich, D8375), 0.5 µM Rotenone/Antimycin A (Sigma Aldrich, R8875/A8674) + 8 µM Hoechst3342 (ThermoFisher, 62249). For the flux assay, 3 baseline measurements were followed by the injection of port A-D, while each injection included 3 measurement cycles. Each measurement cycle consisted of 3 min mixing and 3 min measurement time frames. For normalization, cell counts were measured via Hoechst staining in the Cytation1 with the implemented cell measurement software (Agilent Technologies, Santa Clara, CA, USA). Analysis was conducted in the Seahorse Wave software (RRID:SCR_014526).

Viability assay

NCI-H295R or CD8+ T cells were seeded into black 96-well plate with clear bottom at a density of 2.5×10^4 cells/well or 1×10^4 cells/well, respectively. At the end of the treatment duration, cell viability was detected using CellTiter Glo Assay (Promega) according to the manufacturer's instruction.

Table S1: List of antibodies used for IHC, IC, IF, Flow and WB

**Primary antibodies**					
**method**	**antigen**	**supplier**	**Cat#**	**RRID**	**dilution**
IHC	CD68	abcam	ab955	AB_307338	1:10000
	CD163	abcam	ab182422	AB_2753196	1:200
	CXCL9	abcam	ab202961	AB_3105888	1:150
IC	CD64	abcam	ab288731	AB_3675762	1:50
	MerTK	abcam	ab300136	AB_2936858	1:50
	CD163	R&D Systems	AF1607	AB_354889	1:100
IF	CD68	abcam	ab955	AB_307338	1:100
	CD163	R&D Systems	AF1607	AB_354889	1:100
	C1QA	LSBio	LS-C100911	AB_2067267	1:200
Flow	CD64	abcam	ab288731	AB_3675762	1:100
	CD3	Biolegend	300412	AB_314066	5 µl per million cells in 100 µl staining volume
	CD4	Biolegend	317440	AB_2562912	5 µl per million cells in 100 µl staining volume
	CD8	Biolegend	344718	AB_10551438	5 µl per million cells in 100 µl staining volume
	CXCR3	Biolegend	353704	AB_10983066	10 µl per million cells in 100 µl staining volume
	CD163	R&D Systems	AF1607	AB_354889	2 µl in 100 µl staining volume
	C1QA	LSBio	LS-C100911	AB_2067267	5 µl in 100 µl staining volume
WB	CD163	cell signaling	25121	AB_3675763	1:1000
	CD206	cell signaling	91992	AB_2800175	1:1000
	GR	cell signaling	12041	AB_2631286	1:1000
	MerTK	abcam	ab52968	AB_2143584	1:2000
	C1Q	Novus Biologicals	NBP1-87492	AB_11002443	1:1000
	GAPDH	sigma	G9545	AB_796208	1:10000
**Secondary antibodies**					
AlexaFluor 555 donkey-anti rabbit IgG (H+L)		Invitrogen	A31572	AB_162543	1:500 (IC)
AlexaFluor 488 donkey-anti goat IgG (H+L)		Invitrogen	A11055	AB_2534102	1:500 (IC)<br>1:200 (Flow)
AlexaFluor 647 donkey-anti mouse IgG (H+L)		Invitrogen	A31571	AB_162542	1:1000 (IC)<br>1:200 (Flow)
AlexaFluor 594 donkey-anti rabbit IgG (H+L)		Invitrogen	A21207	AB_141637	1:200 (Flow)
Goat anti-rabbit peroxidase conjugated		Jackson ImmunoResearch	111-035-003	AB_2313567	1:10000 (WB)
Goat anti-mouse peroxidase conjugated		Jackson ImmunoResearch	115-035-003	AB_10015289	1:10000 (WB)

Fig. S1

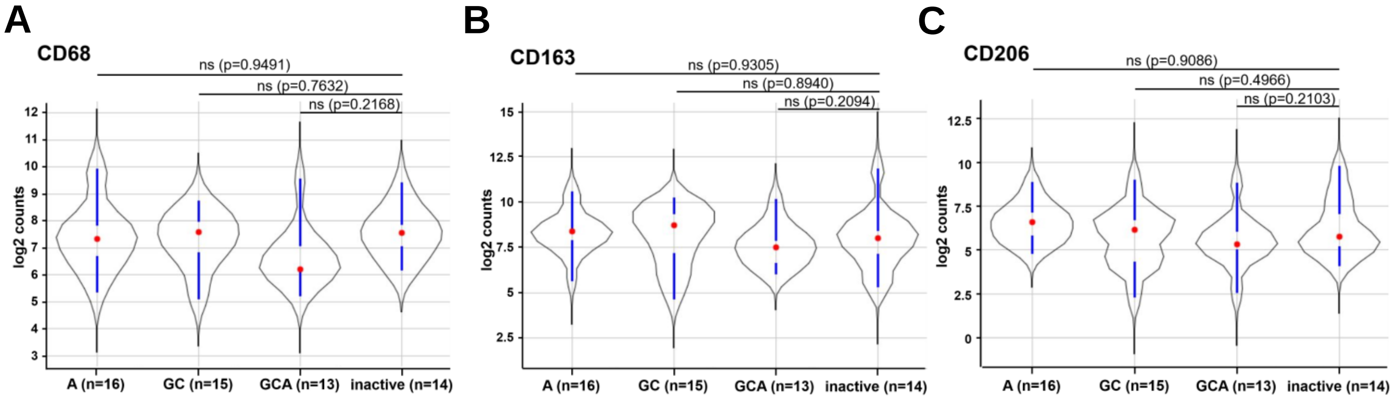
A
CD68
ns (p=0.9491)
ns (p=0.7632)
ns (p=0.2168)
log2 counts
A (n=16)
GC (n=15)
GCA (n=13)
inactive (n=14)
B
CD163
ns (p=0.9305)
ns (p=0.8940)
ns (p=0.2094)
log2 counts
A (n=16)
GC (n=15)
GCA (n=13)
inactive (n=14)
C
CD206
ns (p=0.9086)
ns (p=0.4966)
ns (p=0.2103)
log2 counts
A (n=16)
GC (n=15)
GCA (n=13)
inactive (n=14)

Fig. S2

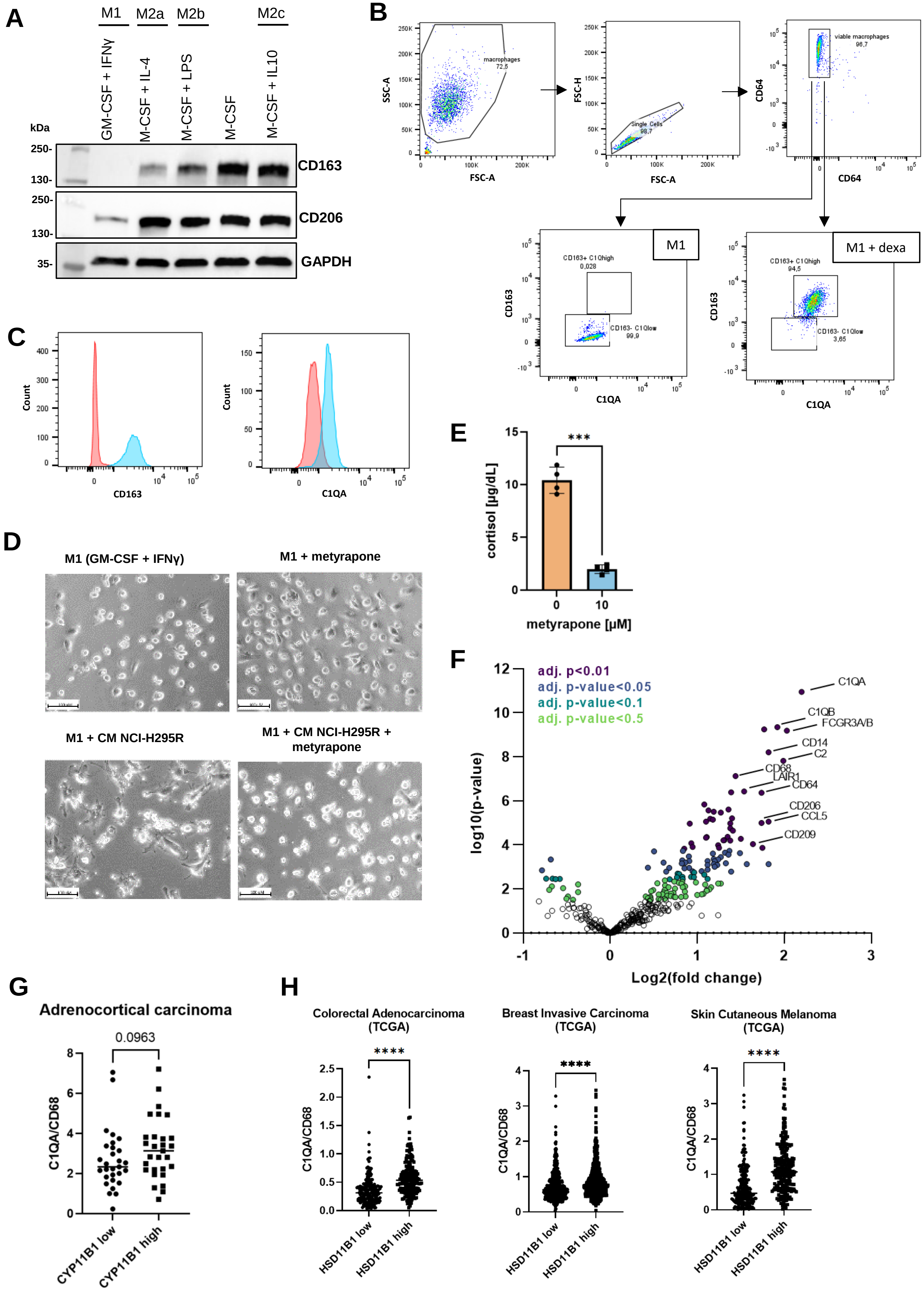
A
M1
M2a
M2b
M2c
GM-CSF + IFNγ
M-CSF + IL-4
M-CSF + LPS
M-CSF
M-CSF + IL10
kDa
250-
130-
CD163
CD206
35-
GAPDH
B
SSC-A
FSC-A
macrophages
72,5
FSC-H
Single Cells
98,7
CD64
viable macrophages
96,7
CD163
C1QA
M1
CD163+ C1Qhigh
0,028
CD163- C1Qlow
99,9
M1 + dexa
CD163+ C1Qhigh
94,5
CD163- C1Qlow
3,65
C
Count
CD163
C1QA
D
M1 (GM-CSF + IFNγ)
M1 + metyrapone
M1 + CM NCI-H295R
M1 + CM NCI-H295R + metyrapone
E
cortisol [µg/dL]
***
metyrapone [µM]
F
adj. p<0.01
adj. p-value<0.05
adj. p-value<0.1
adj. p-value<0.5
C1QA
C1QB
FCGR3A/B
CD14
C2
CD68
LAIR1
CD64
CD206
CCL5
CD209
log10(p-value)
Log2(fold change)
G
Adrenocortical carcinoma
0.0963
C1QA/CD68
CYP11B1 low
CYP11B1 high
H
Colorectal Adenocarcinoma (TCGA)
Breast Invasive Carcinoma (TCGA)
Skin Cutaneous Melanoma (TCGA)
****
C1QA/CD68
HSD11B1 low
HSD11B1 high

Fig. S3

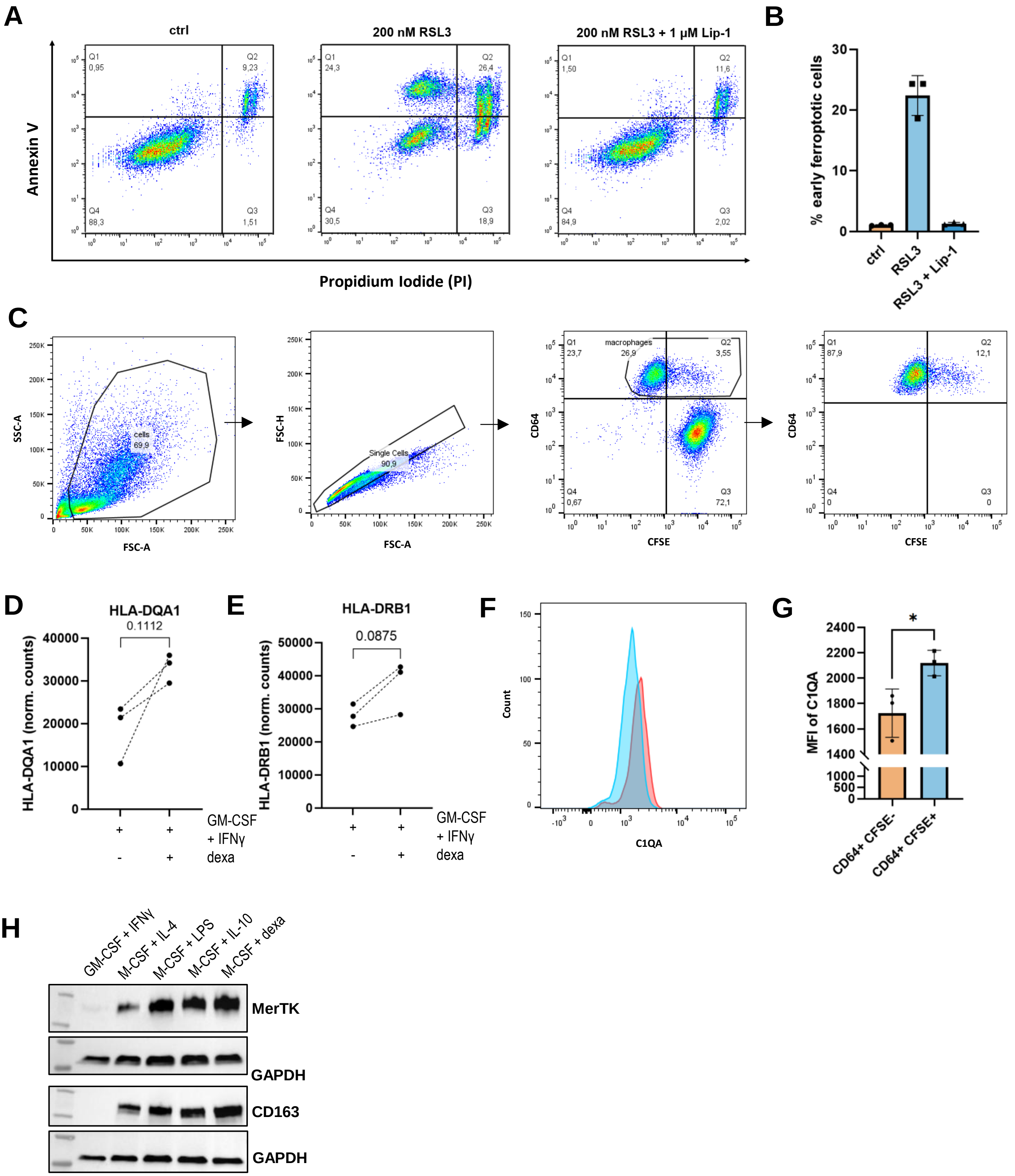

A
ctrl
200 nM RSL3
200 nM RSL3 + 1 µM Lip-1
Annexin V
Propidium Iodide (PI)
Q1 0,95
Q2 9,23
Q4 88,3
Q3 1,51
Q1 24,3
Q2 26,4
Q4 30,5
Q3 18,9
Q1 1,50
Q2 11,6
Q4 84,9
Q3 2,02
B
% early ferroptotic cells
ctrl
RSL3
RSL3 + Lip-1
C
SSC-A
FSC-A
cells 69,9
FSC-H
Single Cells 90,9
CD64
CFSE
macrophages 26,9
Q1 23,7
Q2 3,55
Q4 0,67
Q3 72,1
Q1 87,9
Q2 12,1
Q4 0
Q3 0
D
HLA-DQA1
0.1112
HLA-DQA1 (norm. counts)
E
HLA-DRB1
0.0875
HLA-DRB1 (norm. counts)
GM-CSF + IFNγ
dexa
F
Count
C1QA
G
*
MFI of C1QA
CD64+ CFSE-
CD64+ CFSE+
H
GM-CSF + IFNγ
M-CSF + IL-4
M-CSF + LPS
M-CSF + IL-10
M-CSF + dexa
MerTK
GAPDH
CD163
GAPDH

Fig. S4

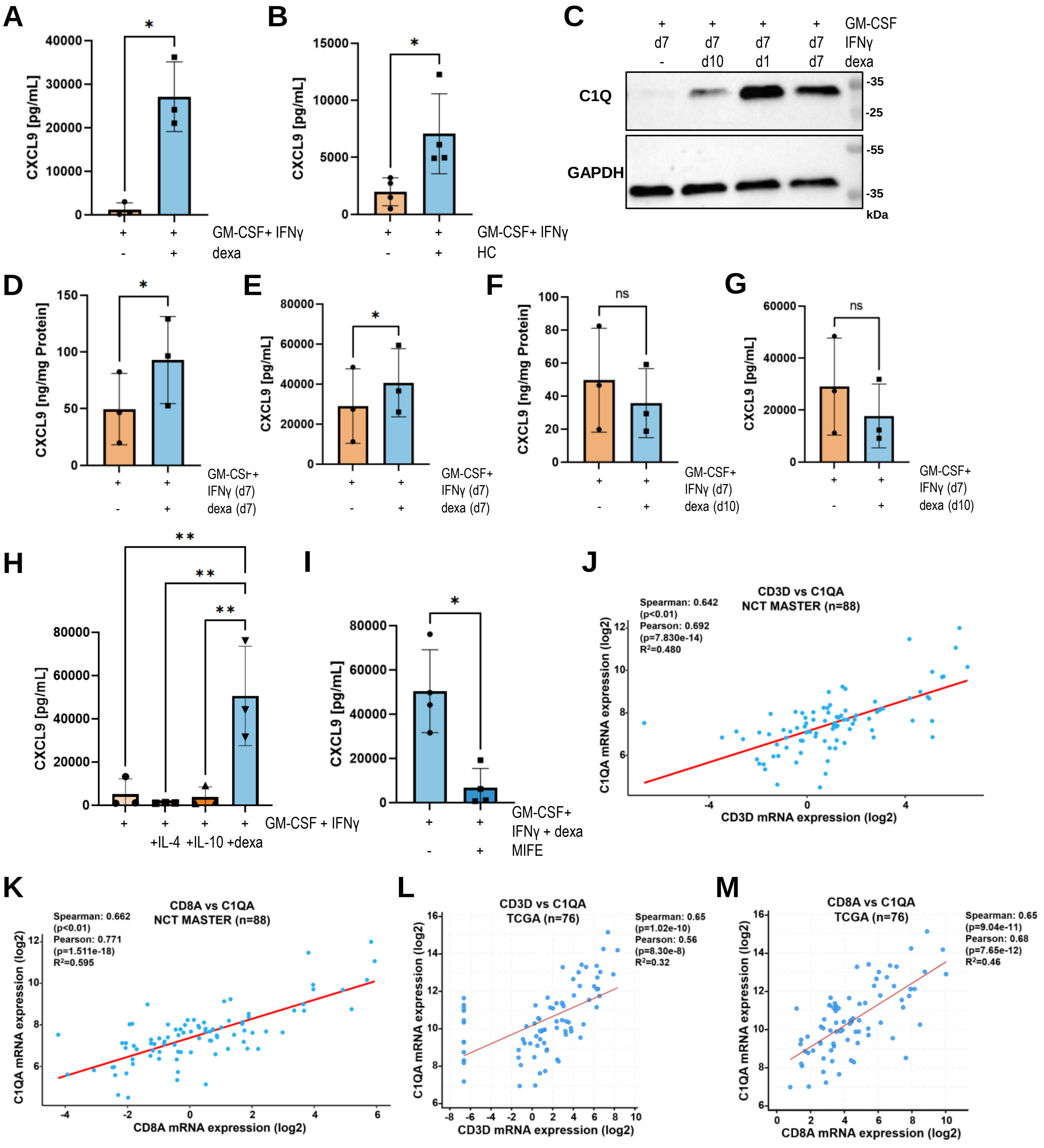
A
B
C
D
E
F
G
H
I
J
K
L
M
CXCL9 [pg/mL]
CXCL9 [ng/mg Protein]
GM-CSF+ IFNγ
dexa
HC
GM-CSF
IFNγ
C1Q
GAPDH
kDa
ns
GM-CSF + IFNγ
+IL-4 +IL-10 +dexa
GM-CSF+
IFNγ + dexa
MIFE
CD3D vs C1QA
NCT MASTER (n=88)
Spearman: 0.642
(p<0.01)
Pearson: 0.692
(p=7.830e-14)
R²=0.480
C1QA mRNA expression (log2)
CD3D mRNA expression (log2)
CD8A vs C1QA
NCT MASTER (n=88)
Spearman: 0.662
(p<0.01)
Pearson: 0.771
(p=1.511e-18)
R²=0.595
CD8A mRNA expression (log2)
CD3D vs C1QA
TCGA (n=76)
Spearman: 0.65
(p=1.02e-10)
Pearson: 0.56
(p=8.30e-8)
R²=0.32
CD8A vs C1QA
TCGA (n=76)
Spearman: 0.65
(p=9.04e-11)
Pearson: 0.68
(p=7.65e-12)
R²=0.46

Fig. S5

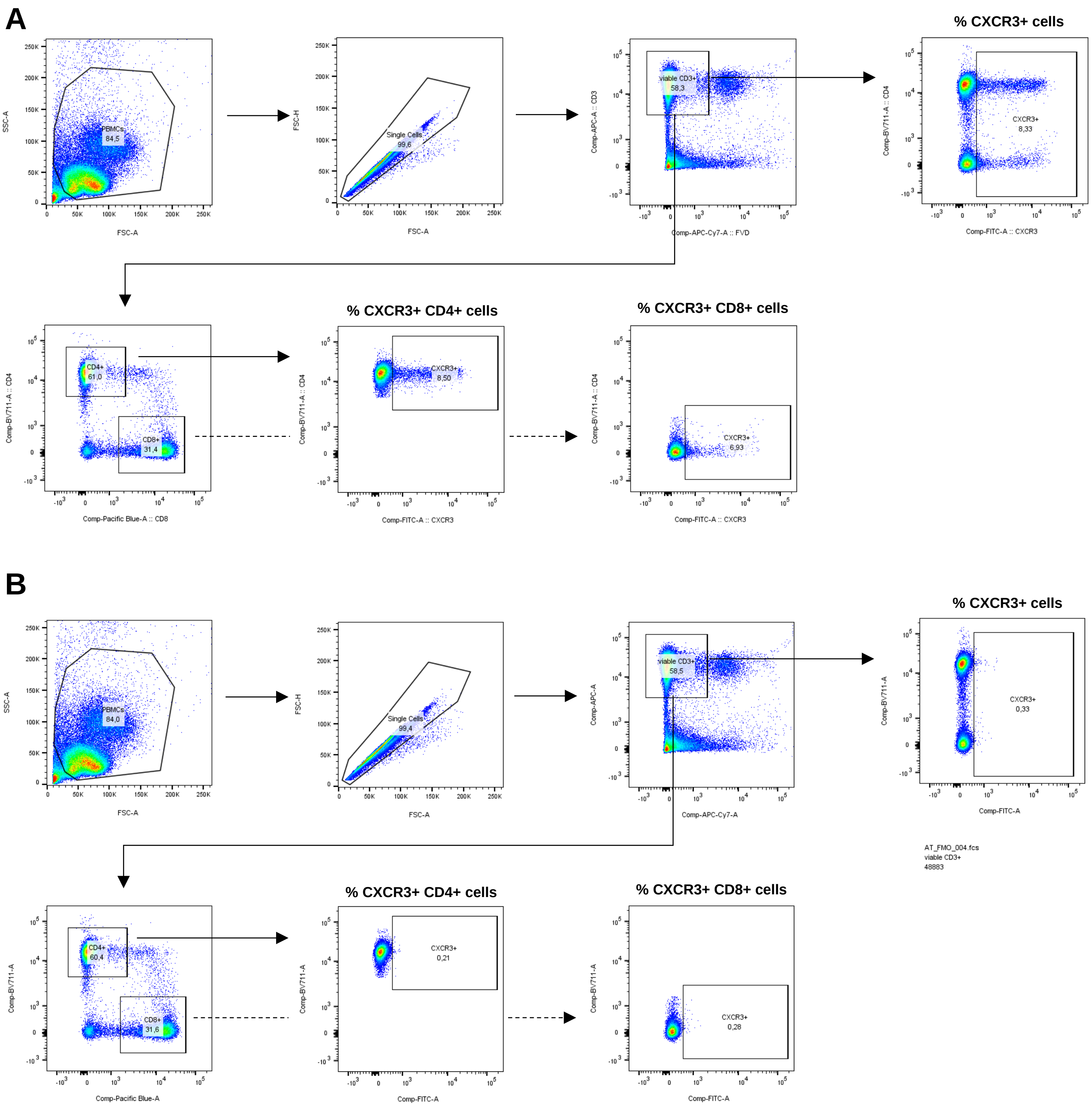

A
% CXCR3+ cells
PBMCs
84,5
SSC-A
FSC-A
Single Cells
99,6
FSC-H
viable CD3+
58,3
Comp-APC-A :: CD3
Comp-APC-Cy7-A :: FVD
CXCR3+
8,33
Comp-BV711-A :: CD4
Comp-FITC-A :: CXCR3
% CXCR3+ CD4+ cells
% CXCR3+ CD8+ cells
CD4+
61,0
CD8+
31,4
Comp-Pacific Blue-A :: CD8
CXCR3+
8,50
CXCR3+
6,93
B
% CXCR3+ cells
PBMCs
84,0
Single Cells
99,4
viable CD3+
58,5
Comp-APC-A
Comp-APC-Cy7-A
CXCR3+
0,33
Comp-BV711-A
Comp-FITC-A
AT_FMO_004.fcs
viable CD3+
48883
% CXCR3+ CD4+ cells
% CXCR3+ CD8+ cells
CD4+
60,4
CD8+
31,6
Comp-Pacific Blue-A
CXCR3+
0,21
CXCR3+
0,28

Fig. S6

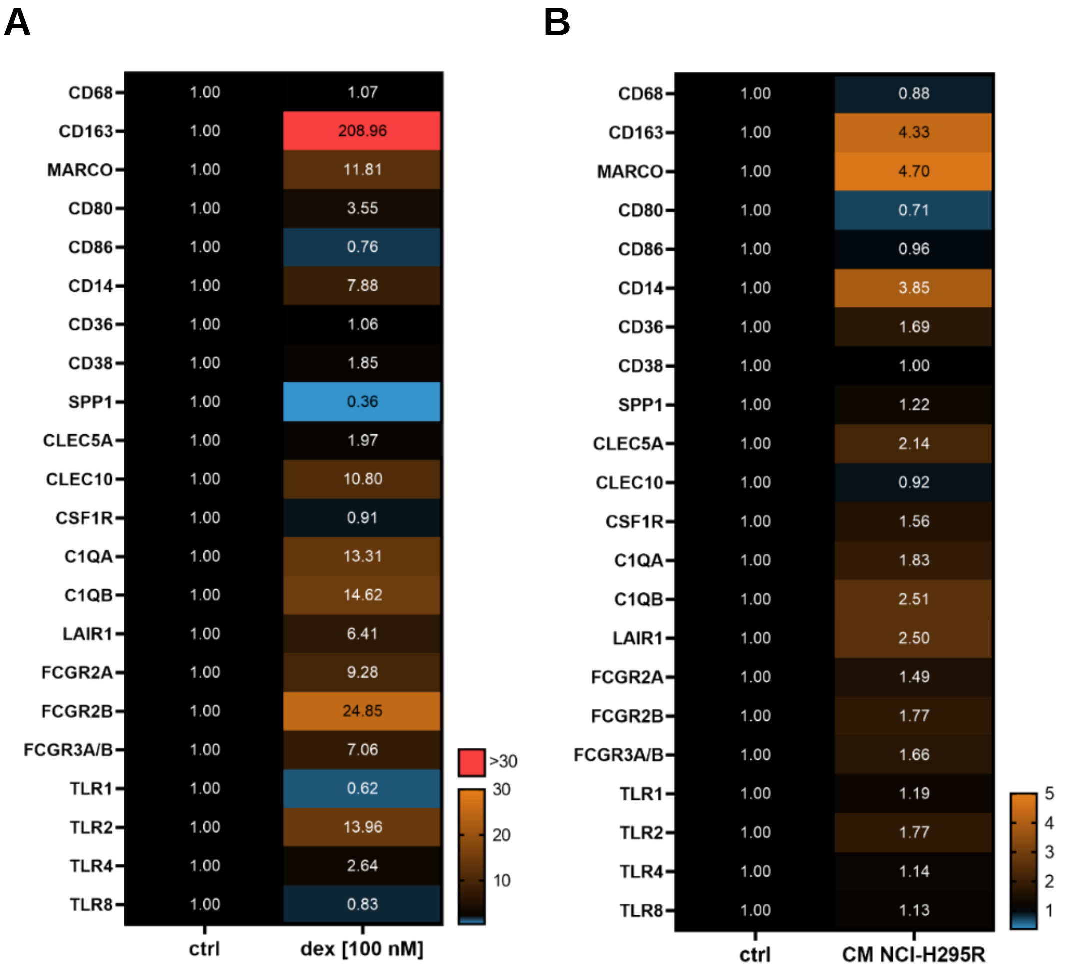
A
CD68
CD163
MARCO
CD80
CD86
CD14
CD36
CD38
SPP1
CLEC5A
CLEC10
CSF1R
C1QA
C1QB
LAIR1
FCGR2A
FCGR2B
FCGR3A/B
TLR1
TLR2
TLR4
TLR8
ctrl
dex [100 nM]
1.00
1.07
208.96
11.81
3.55
0.76
7.88
1.06
1.85
0.36
1.97
10.80
0.91
13.31
14.62
6.41
9.28
24.85
7.06
0.62
13.96
2.64
0.83
>30
30
20
10
B
CD68
CD163
MARCO
CD80
CD86
CD14
CD36
CD38
SPP1
CLEC5A
CLEC10
CSF1R
C1QA
C1QB
LAIR1
FCGR2A
FCGR2B
FCGR3A/B
TLR1
TLR2
TLR4
TLR8
ctrl
CM NCI-H295R
1.00
0.88
4.33
4.70
0.71
0.96
3.85
1.69
1.00
1.22
2.14
0.92
1.56
1.83
2.51
2.50
1.49
1.77
1.66
1.19
1.77
1.14
1.13
5
4
3
2
1

Fig. S7

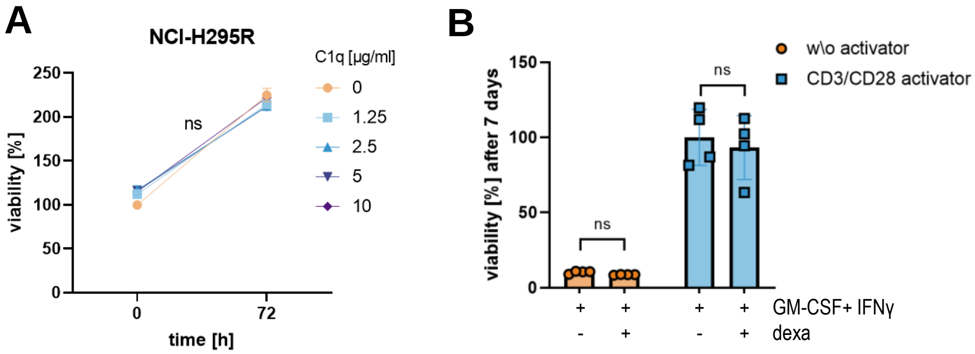
A
NCI-H295R
C1q [µg/ml]
0
1.25
2.5
5
10
ns
viability [%]
250
200
150
100
50
0
0
72
time [h]
B
w\o activator
CD3/CD28 activator
viability [%] after 7 days
150
100
50
0
ns
ns
GM-CSF+ IFNγ
dexa

Fig. S8

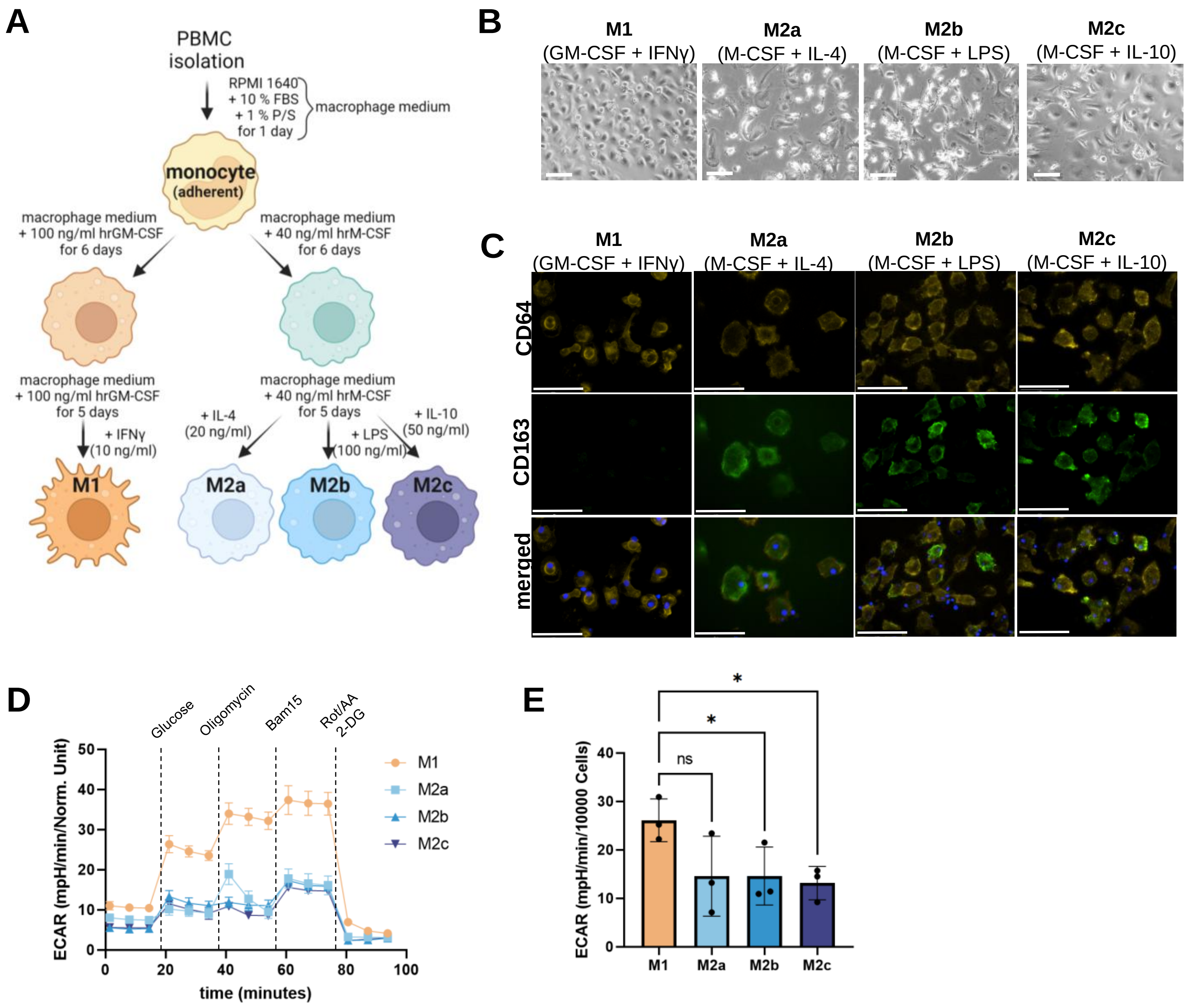
A
PBMC isolation
RPMI 1640
+ 10 % FBS
+ 1 % P/S
for 1 day
macrophage medium
monocyte
(adherent)
macrophage medium
+ 100 ng/ml hrGM-CSF
for 6 days
macrophage medium
+ 40 ng/ml hrM-CSF
for 6 days
macrophage medium
+ 100 ng/ml hrGM-CSF
for 5 days
macrophage medium
+ 40 ng/ml hrM-CSF
for 5 days
+ IFNγ
(10 ng/ml)
+ IL-4
(20 ng/ml)
+ LPS
(100 ng/ml)
+ IL-10
(50 ng/ml)
M1
M2a
M2b
M2c
B
M1
(GM-CSF + IFNγ)
M2a
(M-CSF + IL-4)
M2b
(M-CSF + LPS)
M2c
(M-CSF + IL-10)
C
CD64
CD163
merged
D
Glucose
Oligomycin
Bam15
Rot/AA
2-DG
ECAR (mpH/min/Norm. Unit)
time (minutes)
0
10
20
30
40
50
60
80
100
E
ECAR (mpH/min/10000 Cells)
ns
*
*

Figure S1: (A-C) Violin plots displaying the total tumoral expression of (A) *CD68*, (B) *CD163* and (C) *CD206* determined by Nanostring nCounter expression analysis in a total of 58 ACC tumors, stratified by clinical hormone excess; A=androgen excess; GC=glucocorticoid excess; GCA=glucocorticoid and androgen excess. Red dots represent medians, blue lines connect lower adjusted value and lower quartile and upper adjusted value and upper quartile, respectively. FDR adjusted p-values were calculated using the Nanostring nSolver software and Benjamin&Yekulti correction.

Figure S2: (A) Expression of the macrophage marker CD163, CD206 according to differentiation status detected by western blot. **(B)** Expression of cell surface (CD163) and intracellular (C1QA) macrophage marker assessed by flow cytometry in dexamethasone treated or control macrophages exposed to IFNγ. (**C**) Expression levels of both markers are shown in blue histograms for dexamethasone exposed macrophages and red for control macrophages. **(D)** Morphology of MΦ after exposure to NCI-H295R CM or control as indicated. Representative pictures are shown. Scale bar: 100 µm. CM=conditioned medium. **(E)** Total cortisol in supernatants of NCI-H295R after treatment with metyrapone (10 µM) or DMSO control. Cortisol was measured by a chemiluminescence immunoassay (DiaSorin; REF:313261). N=4 biological replicates. **(F)** Nanostring nCounter analysis of RNA isolated from 58 ACCs. Tumors were classified according to tumoral *CD163* expression into high and low expression; the median was used as cutoff. Adjusted p-values were calculated using Benjamin&Yekulti correction. CM=conditioned medium. **(G)** *C1QA/CD68* gene expression ratio in ACC tumor samples. Tumors were classified according to tumoral *CYP11B1* expression into high and low expression; the median was used as cutoff. Significances were calculated using Mann-Whitney-U-Test. (**H**) *C1QA/CD68* gene expression ratio in Colorectal Adenocarcinoma, Breast Invasive Carcinoma and Skin Cutaneous Melanoma. Gene expression data was obtained from the TCGA dataset. Tumors were classified according to tumoral *HSD11B1* expression into high and low expression; the median was used as cutoff. Significances were calculated using Mann-Whitney-U-Test.

Figure S3: (A) Representative flow cytometry plots and **(B)** quantification of AnnexinV+/PI- populations (early ferroptotic cells) after treatment with the ferroptosis inducer RSL3 (200 nM), RSL3 and Lip-1 (ferroptosis inhibitor, 1 µM) or DMSO control. **(C)** Gating strategy for in vitro phagocytosis assay. Doublet signals were excluded and macrophages were identified by CD64 positivity. Phagocytosis was assessed as the frequency of CFSE+ CD64+ events out of CD64+ events. **(D-E)** RNA expression of the HLA class II genes (F) *HLA-DQA1* and (G) *HLA-DQR1* in macrophages exposed to dexamethasone or control. N=3 biological replicates are shown in Mean +/- SD. Statistical analysis was performed using paired t-test. **(F)** Expression levels of C1QA in CD64+ macrophages after phagocytosis. Expression levels are shown in blue histograms for CFSE- CD64+ macrophages and in red for CFSE+ CD64+ macrophages**.** One representative example is shown**. (G)** Median fluorescence intensity of C1QA is displayed as mean ± SD of three experiments performed using three separate healthy human donors. Groups were compared using unpaired t-test **(H)** Expression of CD163 and MerTK in macrophages according to their differentiation status detected by western blot.

Figure S4: (A) Absolute CXCL9 concentrations in the supernatant of MΦ exposed to GM-CSF+IFNγ vs GM-CSF+IFNγ and dexamethasone (100 nM). N=3 biological replicates from independent healthy blood donors are shown in mean +/- SD. Differences were compared using paired t-test. **(B)** Absolute CXCL9 concentrations in the supernatant of MΦ exposed to GM-CSF+IFNγ vs GM-CSF+IFNγ and hydrocortisone (500 ng/ml). N=3 biological replicates from independent healthy blood donors are shown in mean +/- SD. Differences were compared using paired t-test. **(C)** C1Q protein expression in MΦ treated with GM-CSF (from day 1 onwards), IFNγ (from day 7 onwards) and dexamethasone (100 nM; added on varying timepoints as indicated). **(D-E)** Normalized and (E) absolute CXCL9 concentrations in the supernatant of MΦ exposed to GM-CSF+IFNγ vs GM-CSF+IFNγ and dexamethasone (100 nM; added from day 7 onwards). N=3 biological replicates are shown in Mean +/- SD. Statistical analysis was performed using paired t-test. **(F-G)** Normalized and (G) absolute CXCL9 concentrations in the supernatant of MΦ exposed to GM-CSF+IFNγ vs GM-CSF+IFNγ and dexamethasone (100 nM; added from day 10 onwards). N=3 biological replicates are shown in Mean +/- SD. Statistical analysis was performed using paired t-test. (**H**) Absolute CXCL9 concentrations in the supernatant of MΦ exposed to GM-CSF+IFNγ vs GM-CSF+IFNγ and IL-4 (20 ng/ml), IL-10 (50 ng/ml) or dexamethasone (100 nM). N=3 biological replicates from independent healthy blood donors are shown in mean +/- SD. Groups were compared using one-way ANOVA with Tukey's multiple comparisons test. (**I**) Absolute CXCL9 concentrations in the supernatant of MΦ exposed to GM-CSF+IFNγ vs GM-CSF+IFNγ and mifepristone (10 µM). N=4 biological replicates from independent healthy blood donors are shown in mean +/- SD. Differences were compared using paired t-test **(J-K)** Correlation of *C1QA* and (J) *CD3D* or (K) *CD8A* expression in ACC tumours within the NCT MASTER dataset. N=88. **(L-M)** Correlation of *C1QA* and (L) *CD3D* or (M) *CD8A* expression in ACC tumours within the TCGA dataset. N=76.

Figure S5: (A) Representative flow cytometry gating strategy for identifying $CD3^+$ $CD8^+$ $CXCR3^+$ T cells and $CD3^+$ $CD4^+$ $CXCR3^+$ T cells in PBMCs of ACC patients. Data was obtained from a healthy blood donor. Cells were first gated on live CD3+ cells using a fixable viability dye (ThermoFisher), followed by gating on CD4+ and $CD8^+$ subsets, and finally CXCR3 expression. (**B**) Flow cytometry gating strategy for CXCR3 FMO (fluorescence minus one) controls used to set gates and accurately distinguish positive from negative populations. Data was obtained from one healthy blood donor.

Figure S6: (A-B) Heatmap of macrophage-marker genes differentially expressed after exposure to (A) dexamethasone or (B) CM of NCI-H295R cells compared to M1-polarized macrophages.

Figure S7: (**A**) Viability of the ACC cell lines NCI-H295R and JIL-2266 after 48-hour treatment with different concentrations of human purified C1q or DMSO control. To test for the effects of different C1q concentrations on cell growth, a two-way ANOVA was performed. **(B)** Viability of CD8+ T cells after 7 days of culture in the CM of MΦ polarized with GM-CSF+IFNγ or GM-CSF+IFNγ+dexamethasone, respectively, with or without the addition of CD3/CD28 activator. N=4 biological replicates are shown in Mean +/- SD. Statistical analysis was performed using paired t-tests.

Figure S8: (A) Overview of the *in vitro* differentiation and polarization of primary human monocytes. Created in BioRender. Triebig, A. (2025) https://BioRender.com/z16d389 **(B)** Morphology of differentiated and polarized MΦ by phase contrast microscopy according to the differentiation status indicated. Representative pictures are shown. Scale bar: 100 µm. **(C)** Expression of CD64 and CD163 across different MΦ phenotypes. Representative pictures are shown. Scale bar: 100 µm. **(D)** Extracellular acidification rate (ECAR) from metabolic flux assay of polarized MΦ. Representative graph is shown in Mean +/- SEM for technical replicates. **(E)** Quantified normalized ECAR values for glycolysis. N=3 biological replicates are shown in Mean +/- SD. Statistical comparison to M1 macrophages was performed using one-way ANOVA followed by Šidák's post-hoc test.
